# Mitochondrial impairment increases FL-PINK1 levels by calcium-dependent gene expression^☆^

**DOI:** 10.1016/j.nbd.2013.10.021

**Published:** 2014-02

**Authors:** Rubén Gómez-Sánchez, Matthew E. Gegg, José M. Bravo-San Pedro, Mireia Niso-Santano, Lydia Alvarez-Erviti, Elisa Pizarro-Estrella, Yolanda Gutiérrez-Martín, Alberto Alvarez-Barrientos, José M. Fuentes, Rosa Ana González-Polo, Anthony H.V. Schapira

**Affiliations:** a Centro de Investigación Biomédica en Red sobre Enfermedades Neurodegenerativas (CIBERNED), Departamento de Bioquímica y Biología Molecular y Genética, Universidad de Extremadura, F. Enfermería y Terapia Ocupacional, 10003 Cáceres, Spain; b Department of Clinical Neurosciences, Institute of Neurology, University College London, London NW3 2PF, UK; c INSERM, U848, Institut Gustave Roussy, Université Paris Sud, Paris 11, F-94805 Villejuif, France; d Servicio de Técnicas Aplicadas a las Biociencias, Universidad de Extremadura, 06071 Badajoz, Spain

**Keywords:** SH-SY5Y, CCCP, Parkinson's disease, PINK1, Calcium, Mitophagy

## Abstract

Mutations of the *PTEN-induced kinase 1 (PINK1)* gene are a cause of autosomal recessive Parkinson's disease (PD). This gene encodes a mitochondrial serine/threonine kinase, which is partly localized to mitochondria, and has been shown to play a role in protecting neuronal cells from oxidative stress and cell death, perhaps related to its role in mitochondrial dynamics and mitophagy. In this study, we report that increased mitochondrial PINK1 levels observed in human neuroblastoma SH-SY5Y cells after carbonyl cyanide m-chlorophelyhydrazone (CCCP) treatment were due to de novo protein synthesis, and not just increased stabilization of full length PINK1 (FL-PINK1). PINK1 mRNA levels were significantly increased by 4-fold after 24 h. FL-PINK1 protein levels at this time point were significantly higher than vehicle-treated, or cells treated with CCCP for 3 h, despite mitochondrial content being decreased by 29%. We have also shown that CCCP dissipated the mitochondrial membrane potential (Δψm) and induced entry of extracellular calcium through L/N-type calcium channels. The calcium chelating agent BAPTA-AM impaired the CCCP-induced PINK1 mRNA and protein expression. Furthermore, CCCP treatment activated the transcription factor c-Fos in a calcium-dependent manner. These data indicate that PINK1 expression is significantly increased upon CCCP-induced mitophagy in a calcium-dependent manner. This increase in expression continues after peak Parkin mitochondrial translocation, suggesting a role for PINK1 in mitophagy that is downstream of ubiquitination of mitochondrial substrates. This sensitivity to intracellular calcium levels supports the hypothesis that PINK1 may also play a role in cellular calcium homeostasis and neuroprotection.

## Highlights

• CCCP increases FL-PINK1 by higher gene expression, even after mitophagy induction.
• The increase in *PINK1* gene expression is dependent on calcium-mediated signaling.
• CCCP produces extracellular calcium entry through L- and N-VDCCs.
• CCCP activates the transcription factor c-Fos in a calcium-dependent manner.
• CCCP induces mitophagy by calcium-dependent signaling, but c-Fos-independent.

## Introduction

Parkinson's disease (PD) is a progressive neurodegenerative disorder characterized clinically by bradykinesia, rigidity or tremor, and pathologically by loss of nigrostriatal dopaminergic neurons and the presence of cytoplasmic protein inclusions known as Lewy bodies. The etiology of PD is still unknown, but genetic and possibly environmental factors are thought to be involved (Duvoisin, 1999, Williams et al., 1999). Mitochondrial dysfunction has consistently been implicated in the pathogenesis of PD (Langston et al., 1983, Schapira et al., 1989), and different proteins associated with familial PD, such as PTEN-induced kinase 1 (PINK1), Parkin, DJ-1, LRRK2 and α-synuclein, have been reported to localize to mitochondria and affect function (Bonifati et al., 2003, Devi et al., 2008, Narendra et al., 2008, Papkovskaia et al., 2012, Silvestri et al., 2005, Valente et al., 2004).

Mutations in the *PINK1* gene are responsible for autosomal recessive familial PD (Valente et al., 2004). PINK1 is a 581 amino acid protein ubiquitously transcribed and encodes a serine/threonine kinase, showing high homology with the Ca^2 +^/calmodulin kinase family. Also, PINK1 contains a N-terminal mitochondrial targeting sequence and a C-terminal autoregulatory domain (Beilina et al., 2005, Silvestri et al., 2005, Sim et al., 2006) is predominantly localized to mitochondria, but also is present in the cytosol (Haque et al., 2008, Valente et al., 2004, Weihofen et al., 2008, Zhou et al., 2008). Full-length PINK1 (FL-PINK1), is approximately 63 kDa, and is transcribed in the nucleus, translated in the cytoplasm and imported intact into mitochondria. PINK1 is then cleaved by the mitochondrial protease PARL (presenilin-associated rhomboid-like) at the inner mitochondrial membrane (Deas et al., 2011, Meissner et al., 2011, Whitworth et al., 2008) to yield two bands of 55 kDa (ΔN-PINK1) and 45 kDa (ΔN^2^-PINK1) (Lin and Kang, 2008, Muqit et al., 2006, Silvestri et al., 2005, Weihofen et al., 2008). The ΔN-PINK1 species is rapidly degraded by the proteasome (Takatori et al., 2008).

Previous reports using cell culture models suggest that PINK1 may play a neuroprotective role under several forms of stress conditions, because the over-expression of wild-type *PINK1*, but not mutant *PINK1*, protects against cell death induced by chemical stressors, such as the neurotoxin 1-methyl-4-phenyl-1,2,3,6-tetrahydropyridine (MPTP) or the proteasomal inhibitor carbobenzoxyl-leucyl-leucyl-leucinal (MG-132) (Haque et al., 2008, Petit et al., 2005, Valente et al., 2004). Several studies in *Drosophila*, mouse and cell culture models, including fibroblasts from patients with *PINK1* mutations (Abramov et al., 2011, Grunewald et al., 2009, Hoepken et al., 2007, Piccoli et al., 2008), suggest that loss of *PINK1* can be associated with functional and morphological mitochondrial effects, oxidative stress and the balance between mitochondrial fission and fusion (Clark et al., 2006, Gautier et al., 2008, Gegg et al., 2009, Gispert et al., 2009, Heeman et al., 2011, Park et al., 2006, Poole et al., 2008, Sandebring et al., 2009, Yang et al., 2008). The mitochondrial dysfunction associated with *PINK1* deficiency has been linked to perturbed mitophagy, a cellular process by which old and damaged mitochondria are engulfed into double membrane vacuoles, called autophagosomes, that then fuse with lysosomes, resulting in autophagolysosomes, where mitochondria are subsequently degraded (Kim et al., 2007, Youle and Narendra, 2011). Loss of Δψm induced by mitochondrial uncouplers, like carbonyl cyanide m-chlorophelyhydrazone (CCCP), is an initial step in the removal of this organelle, initiating fission of the reticular mitochondrial network in the damaged mitochondria (Narendra et al., 2008, Twig et al., 2008). This event inhibits the processing of FL-PINK1 by PARL, leading to the accumulation of FL-PINK1 on the mitochondrial outer membrane (Jin et al., 2010, Matsuda et al., 2010, Narendra et al., 2010b, Vives-Bauza et al., 2010). PINK1 then recruits Parkin to mitochondria via phosphorylation (Kondapalli et al., 2012, Matsuda et al., 2010), whereupon Parkin ubiquitinates mitochondrial proteins such as VDAC and the mitofusins (Gegg et al., 2010, Geisler et al., 2010, Ziviani et al., 2010). The ubiquitination of mitochondrial outer membrane proteins such as the mitofusins leads to their degradation by the proteasome, and is required for mitophagy (Chan et al., 2011, Tanaka et al., 2010).

Loss of PINK1 function results in decreased ATP synthesis by mitochondria, impaired mitochondrial calcium handling and increased oxidative stress in a time-dependent manner (Gautier et al., 2008, Gegg et al., 2009). The impairment of mitochondrial function is coincident with decreased macroautophagy flux (Gegg et al., 2010). Restoration of mitophagy in *PINK1*-deficient cells by exogenous expression of *parkin* results in improved mitochondrial function (Gegg et al., 2010), suggesting that impaired mitophagy may contribute to the mitochondrial dysfunction observed in PD.

Calcium plays a central role in regulating neurotransmitter release. Calcium influx is involved in the release of neurotransmitters by synaptic vesicles (Neher and Sakaba, 2008), synaptic plasticity (Zucker, 1999) and gene transcription (Lyons and West, 2011). Studies have implicated abnormal calcium homeostasis as a factor in the pathogenesis of PD, contributing to mitochondrial oxidative stress (Goldberg et al., 2012, Guzman et al., 2010) and dopaminergic neuronal death (Beal, 1998), or increasing levels of Ca^2 +^-binding proteins calretinin and calbindin to confer some protection (Mouatt-Prigent et al., 1994, Yamada et al., 1990). Calcium regulation involves a range of different transcription factors, including c-Fos (FBJ murine osteosarcoma viral oncogene homolog) (Ng et al., 2012, Premkumar et al., 2000, Thompson et al., 1995, Zhang et al., 2002), CREB (cyclic AMP response element binding protein) (Soriano et al., 2006) and NF-κB (nuclear factor-κB) (Camandola et al., 2005, Furukawa and Mattson, 1998).

Furthermore, previous reports have shown that CCCP produces cytosolic [Ca^2 +^] ([Ca^2 +^]_c_) elevations in different cell models (Babcock et al., 1997, Biswas et al., 1999, Eilam et al., 1990, Herrington et al., 1996, Komori et al., 2010, Lim et al., 2006, Pereira et al., 2008, Yan et al., 2012). This is consistent with the calcium entry from the outside (Ardon et al., 2009) and the inhibition of calcium uptake via the mitochondrial uniporter (mCU), pathway driven by the Δψm.

Several studies indicate that PINK1 participates in mitochondrial calcium homeostasis (Gandhi et al., 2009, Heeman et al., 2011), possibly regulating the mitochondrial permeability transition pore (mPTP) (Akundi et al., 2011, Gautier et al., 2012), mitochondrial Na^+^/Ca^2 +^ exchanger (NCXmito) (Gandhi et al., 2009) or mCU (Marongiu et al., 2009). Isoforms NCX2 and NCX3 may act downstream of PINK1, preventing mitochondrial calcium overload (Wood-Kaczmar et al., 2013).

In the current study, we show that the increase in FL-PINK1 levels seen upon CCCP treatment is not solely due to the stabilization of the protein on the mitochondrial outer membrane and decreased degradation by the proteasome (Jin et al., 2010, Matsuda et al., 2010, Narendra et al., 2008), but also to de novo PINK1 synthesis in a calcium-dependent pathway. Moreover, CCCP induced c-Fos activation through the extracellular calcium entrance by the L-type and N-type voltage-dependent calcium channels (VDCCs).

## Material and methods

### Cell culture

The human SH-SY5Y neuroblastoma cell line was cultured in 1:1 (v/v) DMEM:F12 (Ham) media containing 0.9 g/l glucose and supplemented with 10% fetal bovine serum, 1 mM sodium pyruvate, non-essential amino acids, and penicillin–streptomycin. The cells were seeded at a density of 2 × 10^6^ in a 75-cm^2^ tissue culture flask (Corning, New York, NY) and incubated at 37°C under saturating humidity in 5% CO_2_/95% air.

### Treatments

Cells were plated at a density of 1 × 10^6^ cells/ml, and treated with CCCP (10 μM, Sigma-Aldrich, St. Louis, MO) or an equivalent volume of ethanol (vehicle control, Sigma-Aldrich, St. Louis, MO) (Gegg et al., 2010). For the inhibition of transcription and translation, actinomycin D (Act. D) (5 μg/ml, Fisher Scientific, Waltham, MA) and cycloheximide (CHX) (100 μg/ml, Sigma-Aldrich, St. Louis, MO) were employed, respectively. The ubiquitin-proteasome system (UPS) and autophagy were inhibited with MG-132 (5 μM, Tocris Bioscience, Bristol, UK), and bafilomycin A1 (Baf. A1) (100 nM, Sigma-Aldrich, St. Louis, MO), respectively. BAPTA-AM (5 μM, Molecular Probes, Life Technologies, Carlsbad, CA) and EGTA (500 μM, Sigma-Aldrich, St. Louis, MO) were used to chelate intracellular calcium and extracellular calcium, respectively. To inhibit the chaperone Hsp90, cells were incubated with 17-AAG (1 μM, Calbiochem, Merck KGaA, Darmstadt, Germany). Cells were treated with all the inhibitors 1 h prior to CCCP, except 17-AAG, which was added 3 h before recovering the cells because of its toxicity. For the calcium live-cell imaging, we stimulated SH-SY5Y cells with these compounds just before the measurement: thapsigargin (1 μM, Sigma-Aldrich, St. Louis, MO), rotenone (10 μM, Sigma-Aldrich, St. Louis, MO) and Baf. A1 (100 nM) to deplete calcium stores (endoplasmic reticulum (ER), mitochondria and lysosomes, respectively); nifedipine (10 μM, Sigma-Aldrich, St. Louis, MO) and ω-conotoxin GVIA (ω-CTX) (2 μM, Alomone Labs, Jerusalem, Israel), L- and N-type VDCC blockers, respectively. In addition, cells were incubated with the following treatments for 24 h: calcium ionophore ionomycin (0.1–2 μM, Sigma-Aldrich, St. Louis, MO) and L-type VDCC agonist Bay K 8644 (0.25–5 μM, Tocris Bioscience, Bristol, UK).

### Citrate synthase activity

Following treatments, cells were washed with PBS and lysed in 0.25% (v/v) Triton X-100 (Sigma-Aldrich, St. Louis, MO) in PBS supplemented with protease and phosphatase inhibitors. Debris was removed by centrifugation and citrate synthase (CS) activity was measured by following the oxidation of 5,5′-Dithiobis(2-nitrobenzoic acid) (Sigma-Aldrich, St. Louis, MO) in a spectrophotometer (absorbance at 412 nm) over time at 30°C in the presence of acetyl co-enzyme A (Sigma-Aldrich, St. Louis, MO) and oxaloacetate (Sigma-Aldrich, St. Louis, MO) (Clark and Land, 1974). Protein concentration was measured based on the bicinchoninic acid (BCA) method, using a Bicinchoninic Acid Kit (Sigma-Aldrich, St. Louis, MO) using bovine serum albumin (BSA) as a standard. The enzyme activity was expressed as nmol/min/mg protein.

### Plasmid transfection

For the over-expression of PINK1 (to compare with the endogenous protein), SH-SY5Y cells were transfected with pCMV6-Neo vector containing full-length wild-type *PINK1* cDNA (Origene), as previously described (Gegg et al., 2009), using FuGENE® 6 Transfection Reagent (Roche, Indianapolis, IN), according to the manufacturer's protocol. mCherry-Parkin (Addgene plasmid 23956) (Narendra et al., 2008), GFP-LC3 (Kabeya et al., 2000) (Dr. Tamotsu Yoshimori gift) and mCherry-GFP-LC3B (Pankiv et al., 2007) (Dr. Terje Johansen gift) constructs were transfected as previously reported (Bravo-San Pedro et al., 2013).

### Transient transfection with *PINK1/c-fos* siRNA

Cells (1.8 × 10^5^ cells/ml) were transfected with a pair of *PINK1* siRNAs (5 nM each), *c-fos* siRNA (50 nM) or 10 nM scrambled control siRNA (Ambion negative control siRNA #1, Enzo Life Sciences, New York, NY) using HiPerfect transfection reagent (Qiagen, Crawley, UK) (for *PINK1* siRNA) or Lipofectamine™ 2000 transfection reagent (Invitrogen, Life Technologies, Carlsbad, CA) (for *c-fos* siRNA). A combination of *PINK1* siRNA pair was used (SI00287931, sense strand: GACGCUGUUCCUCGUUAUGAA; and SI00287924, sense strand: CGGACGCUGUUCCUCGUUAU) (Qiagen, Crawley, UK). The *c-fos* siRNA (SI02781429) had the following sense strand: CCAAUAUUAUACUAAGAAATT (Qiagen, Crawley, UK).

### Quantitative PCR

Total RNA was extracted from SH-SY5Y cells using the RNeasy Mini Kit (Qiagen, Crawley, UK). cDNA was synthesized by the QuantiTect Reverse Transcription Kit (Qiagen, Crawley, UK), adding 500 ng of template RNA. *PINK1* gene expression was measured by quantitative real-time PCR (qPCR) with SYBR Green reagents (Applied Biosystems, Life Technologies, Carlsbad, CA). To normalize the results, housekeeping gene glyceraldehyde 3-phosphate dehydrogenase (*GAPDH*) expression was used. Analysis of relative gene expression was calculated using the comparative threshold (2^− ΔΔCt^) method (Pfaffl, 2001). Primer sequences were performed as previously described (Gegg et al., 2009).

### Western-blotting

Protein was obtained using a lysis buffer composed of 1% (v/v) Triton X-100 in PBS supplemented with protease and phosphatase inhibitors, and quantified according to the CS activity protocol above. Equal amounts of protein (50–75 μg/condition) were resolved by 4–20% SDS-gel electrophoresis and transferred to polyvinylidene fluoride (PVDF) membranes (Hybond P, GE Healthcare, Chalfont St. Giles, UK) according to a partially modified conventional method (Fuentes et al., 2000). Immunodetection included transferring and blocking the membrane with TBST (Tris-buffered saline with Tween 20) buffer containing 10% non-fat dried milk. Blots were probed with antibodies against PINK1 (clone BC100-494, Novus Biologicals, Southpark Way, Littleton, CO), subunit IV of cytochrome c oxidase (COX IV, ab14744, abcam), prohibitin 1 (#2426, Cell Signaling Technology, Beverly, MA), LC3B (#2775, Cell Signaling Technology, Beverly, MA), p-c-Fos (Ser32) (#5348, Cell Signaling Technology, Beverly, MA), c-Fos (#2250, Cell Signaling Technology, Beverly, MA), β-actin (ab8227, Abcam, Cambridge, UK), α-tubulin (clone TU-02, Santa Cruz Biotechnology, Santa Cruz, CA), Tom20 (clone F-10, Santa Cruz Biotechnology, Santa Cruz, CA), and Lamin A/C (612162, BD Biosciences, Franklin Lakes, NJ). Blots were visualized with respective peroxidase-conjugated secondary antibodies (Bio-Rad, Hercules, CA) and visualized by chemiluminescence using ECL substrate (Pierce, Thermo Fisher Scientific, Rockford, IL). The β-actin and α-tubulin content was established as loading control, Tom20 as mitochondrial loading control and Lamin A/C as nuclear loading control. The density of bands was determinated using ImageJ software (NIH, Bethesda, MD). For quantitative studies, three separate western blots were analyzed.

### Mitochondrial isolation

Cell pellets (1 × 10^7^ cells/condition) were washed with PBS supplemented with protease and phosphatase inhibitors and homogenized with a glass-teflon homogenizer in isolation medium (250 mM sucrose, 1 mM EDTA, 10 mM Tris, pH 7.4, supplemented with protease and phosphatase inhibitors). Nuclei were removed by centrifugation at 1,500 ×*g*. A second centrifugation at 12,000 ×*g* separated cytosolic (supernatant) and mitochondrial fractions, and the pellet was resuspended in isolation medium. Protein concentration was measured using the BCA method, as described in the CS assay.

### Flow cytometry

After exposure to different experimental conditions, SH-SY5Y cells were trypsinized and labeled with different fluorochromes. To measure the intracellular calcium levels, cells were incubated with Fluo-3 AM (1 μM, Molecular Probes, Life Technologies, Carlsbad, CA) for 30 min at 37 °C. Δψm was assessed with tetramethylrhodamine methyl ester perchlorate (TMRM) (25 nM, Molecular Probes, Life Technologies, Carlsbad, CA) for 30 min at 37°C. Propidium iodide (PI) (1 μg/ml, Sigma-Aldrich, St. Louis, MO) was used to determine the cell viability (Gonzalez-Polo et al., 2007). PI-negative cells were considered to be viable. Single-parametric analysis was performed with a Cytomics FC500 MPL Flow Cytometer (Beckman Coulter, Miami, FL), which has an argon ion laser operating at 488 nm, with subsequent data analysis using Summit v4.2 software (DakoCytomation, Fort Collins, CO). All measurements were developed in duplicate (10,000 cells/sample) and in at least three independent experiments.

### Immunofluorescence

To measure Δψm by this technique, cells were washed with PBS and incubated with TMRM for 30 min at 37°C. For the detection of endogenous PINK1 and mitochondrial network, cells were seeded on coverslips, fixed with paraformaldehyde (4% w:v), permeabilized with Triton X-100 solution (Triton X-100 0.2% in PBS) and stained with PINK1 (clone BC100-494, Novus Biologicals, Southpark Way, Littleton, CO) and Tom20 (clone F-10, Santa Cruz Biotechnology, Santa Cruz, CA), then cells were incubated with the respective Alexa Fluor 488 anti-rabbit, 488 anti-mouse and 568 anti-mouse secondary antibodies (Molecular Probes, Life Technologies, Carlsbad, CA). Mitochondrial morphology was determined using ImageJ software. Mitochondria were scored for their circularity, with 0 being a straight line, and 1.0 a perfect circle. In this sense, mitochondria were designated fragmented when the circularity was between 0.60 and 1.00 and tubular when between 0.00 and 0.60. For the transcription factor staining, cells were prepared in the same way, using p-c-Fos (Ser32) (#5348, Cell Signaling Technology, Beverly, MA) and c-Fos (#2250, Cell Signaling Technology, Beverly, MA). The nuclear morphology and chromatin condensation were labeled with Hoechst 33342 (Ho) (2 μM; Sigma-Aldrich, St. Louis, MO). Cells with condensed nuclei were considered not to be viable. TMRM and Ho staining were analyzed using an inverted fluorescence microscope (IX51, Olympus, Tokyo, Japan) equipped with a camera (DP70, Olympus, Tokyo, Japan). The colocalization of PINK1 with mitochondria and nuclear c-Fos, images were taken with a confocal scanning laser (Nikon A1, Tokyo, Japan) coupled to an inverted microscope (Eclipse T*i*, Nikon, Tokyo, Japan). The quantitative measurement of the fluorescence signal was performed using ImageJ software. For the colocalization studies, the samples were quantified using the JaCoP plug-in for ImageJ, based in Mander's coefficient (Bolte and Cordelieres, 2006), analyzing at least 30 cells for each condition. For the other measurements, we counted at least 200 cells per condition. In the nuclear colocalization experiments, pictures were converted into “6_shades” LUT, setting in ImageJ software.

### Measurement of intracellular calcium

To perform calcium measurements, cells were seeded at a density of 1 × 10^6^ cells/ml on coverslips of 14 mm. Cells were loaded with Fura-2 AM (5 μM, Molecular Probes, Life Technologies, Carlsbad, CA) for 45 min at 37°C in Locke's K25 buffer that contained 137 mM NaCl, 5 mM KCl, 4 mM NaHCO_3_, 2 mM CaCl_2_, 1 mM MgCl_2_, 5 mM glucose, and 10 mM HEPES, pH 7.4, and then washed three times with Locke's K25 buffer at 37 °C to remove extracellular Fura-2 AM. Fura-2 was excited alternatively at 340 nm and 380 nm, and emitted at 510 nm using an illumination system (MT20, Olympus, Tokyo, Japan). The ratio of fluorescent signals obtained at 340 nm and 380 nm excitation wavelengths was recorded at 2 second intervals during 10–15 min, depending on the experiment. F340/F380 ratio was used to represent the [Ca^2 +^]_i_ in the cells. The fluorescence of Fura-2-loaded SH-SY5Y cells was monitored and acquired with a CCD camera (Hammamatsu Orca-ER, Shizouka, Japan) for ratiometric imaging, mounted on an inverted microscope (IX81, Olympus, Tokyo, Japan). Images were analyzed using the software Cell^R (Olympus, Tokyo, Japan). Fields of cells and regions of interest (ROIs) were chosen based on homogenous and equal cell densities. For the calcium channel blockers (nifedipine and ω-CTX), cells were treated before they were measured as was described in treatments. All the experiments were performed at 37°C.

### Nuclear isolation

Nuclear and cytoplasmic fractions were obtained from cell lysates (1 × 10^7^ cells/condition), using the EpiQuik™ Nuclear Extration Kit (Epigentek, Farmingdale, NY). Protein concentration was determined by the BCA method assay, how it is detailed in the CS measurement.

### Statistical analyses

Each experiment was repeated at least three times, with a satisfactory correlation between the results of individual experiments. The data shown are those of a representative experiment; each group was the average of three to four culture dishes. In each experiment, the differences between groups were assessed by appropriate statistical methods. Specifically, statistical significance was evaluated with two-tailed unpaired Student's *t*-test and ANOVA test, and all comparisons with p value less than 0.05 (p < 0.05) were considered statistically significant. The data are expressed as the mean ± SEM. All data were analyzed with SPSS Statistics 20 (IBM, Chicago, IL) for Windows.

## Results

### Mitochondrial damage and autophagy induction by CCCP

**Fig. 1:**
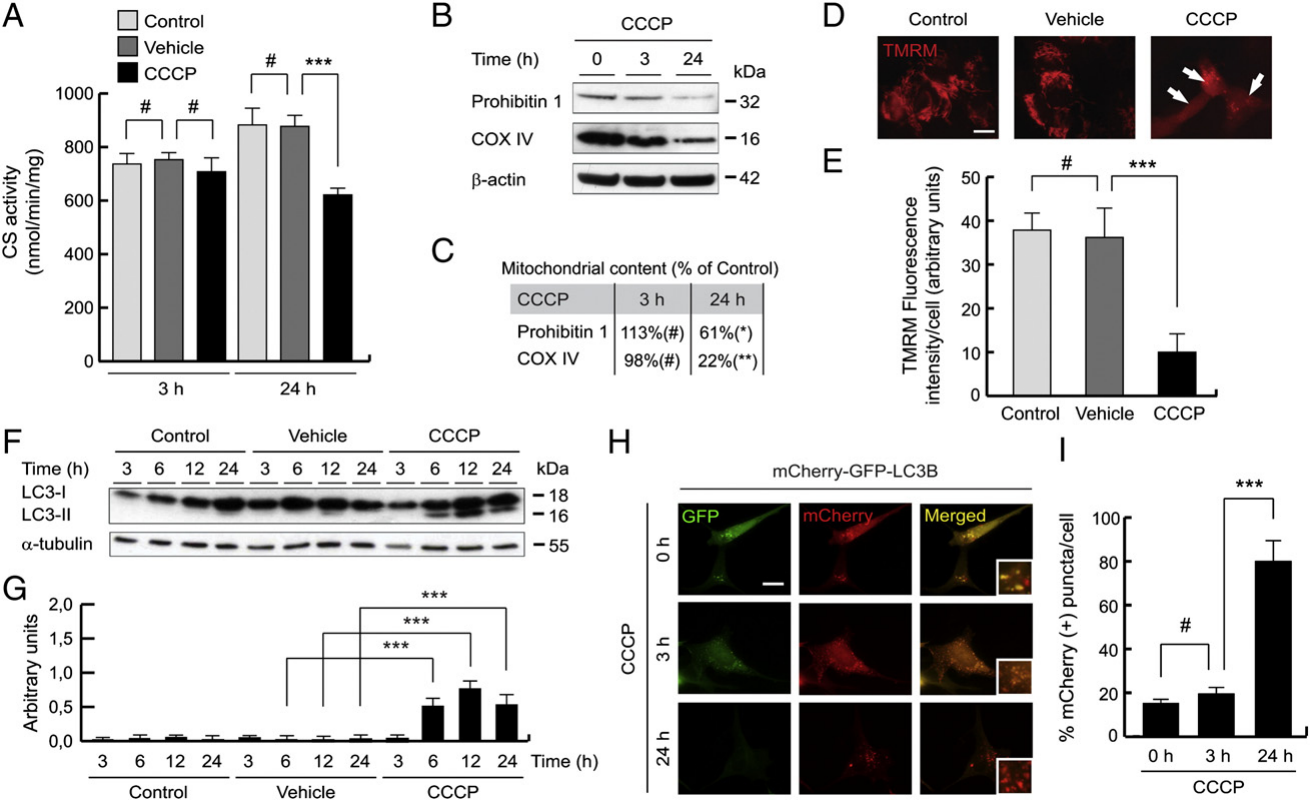
Mitochondrial damage and autophagy induction in SH-SY5Y CCCP-treated cells. (A) SH-SY5Y cells were exposed with 10 μM CCCP, with vehicle (0.05% (v/v) ethanol) or without any treatment (control), lysed and CS activity measured. Data are expressed as nmol/min/mg protein (#p > 0.05; ***p ≤ 0.001). (B and C) SH-SY5Y cells were treated with 10 μM CCCP for 0, 3 or 24 h and lysates separated by SDS-PAGE and Western-blotting performed. Blots were probed with antibodies against two inner mitochondrial membrane proteins, COX IV and prohibitin 1. β-actin was used as a loading control. (B) Representative blot of at least three independent experiments. (C) Densitometry of each band expressed as % of control (#p > 0.05; *p ≤ 0.05; **p ≤ 0.01). (D and E) SH-SY5Y cells were exposed with 10 μM CCCP, with vehicle (0.05% (v/v) ethanol) or without any treatment (control) for 24 h, and stained with TMRM to assess Δψm by immunofluorescence. (D) Representative microphotographs of TMRM stain. Scale bar represents 10 μm. The arrows highlight cells with low Δψm. (E) TMRM fluorescence intensity per cell (in AU) by immunofluorescence (# p > 0.05; *** p ≤ 0.001). (F and G) SH-SY5Y cells were exposed with 10 μM CCCP, with vehicle (0.05% (v/v) ethanol) or without any treatment (control), harvested by trypsinization at different times and lysed. The ratio LC3-II/LC3-I was determined by Western-blotting. α-tubulin expression was used as a loading control. (F) Representative blot of at least three independent experiments. (G) Densitometry of each band expressed in arbitrary units of intensity (***p ≤ 0.001). Molecular mass is indicated in kilodaltons (kDa) next to the blots. Data were expressed as mean ± SEM; n = 3. (H and I) SH-SY5Y cells were transfected with mCherry-GFP-LC3B plasmid for 24 h and treated with 10 μM CCCP for 0, 3 or 24 h and fixed. (H) Representative immunofluorescence microphotographs. Autophagolysosomes and autophagosomes were labeled by red (mCherry-LC3B) and yellow puncta (mCherry-GFP-LC3B), respectively. The boxes highlight the pattern of each condition. (I) Percentages of mCherry (+) puncta per cell (#p > 0.05; ***p ≤ 0.001). Scale bar represents 10 μm.

CCCP is a lipid-soluble weak acid, which acts as a very powerful mitochondrial uncoupling agent that carries protons across the mitochondrial inner membrane, thereby diminishing the proton gradient. To investigate the molecular events in CCCP-induced depolarization, according to previous reports (Narendra et al., 2010a, Narendra et al., 2010b, Vives-Bauza et al., 2010), cells were treated with 10 μM CCCP. The mitochondrial content in SH-SY5Y cells was assessed by measuring the CS activity (Gegg et al., 2010, Wood-Kaczmar et al., 2008). CCCP treatment produced a significant decrease in mitochondrial mass (29.2 ± 3.1%) (mean ± standard deviation [SD]) after 24 h (Fig. 1A), meanwhile a short exposure (3 h) did not result in decreased mitochondrial content. Furthermore, washing away of CCCP after 6 h of treatment, and measuring mitochondrial content 18 h later (24 h after start of treatment), no longer resulted in a significant decrease in mitochondrial content (data not shown). The decrease in CS activity was not due to a failure of the enzyme being imported in to mitochondria because of a lack of mitochondrial membrane potential, as we have previously shown that (a) knockdown of *PINK1* or *parkin* reverses the decrease in CS activity following CCCP treatment and (b) CS loss was greater in SH-SY5Y over expressing *parkin*, when compared to SH-SY5Y cells with endogenous Parkin levels (Gegg et al., 2010).

To further prove that the decrease of CS activity after 24 h of CCCP treatment was due to the loss of mitochondria, we analyzed mitochondrial protein levels by Western-blotting, checking two inner mitochondrial membrane proteins, COX IV and prohibitin 1. CCCP treatment for 24 h caused a 61% reduction in prohibitin 1 and a 22% reduction in COX IV (Figs. 1B and C), reflecting the fall in mitochondrial content. To prove that CCCP produced Δψm reduction, we measured TMRM in SH-SY5Y by immunofluorescence. In this sense, CCCP-treated cells shown a Δψm dissipation at 24 h (10.5 ± 5.1 arbitrary units (AU) of TMRM fluorescence intensity/cell), when compared with untreated cells (37.6 ± 5.1 AU) or vehicle-treated cells (35.6 ± 8.1 AU) (Figs. 1D and E). This Δψm decrease was observed after 1 hour CCCP exposure (data not shown), and we confirmed this result by flow cytometry (data not shown).

To confirm that CCCP is inducing autophagy in our cell model, and thereby the mechanism by which mitochondria are being degraded, we analyzed the autophagic protein LC3. LC3 presents as a cytosolic form (called LC3-I), which can then be conjugated to phosphatidylethanolamine to generate LC3-phosphatidylethanolamine conjugate (LC3-II), that is then recruited to autophagosomes. An increase in LC3-II protein levels can be used as a marker of autophagosome formation. In this sense, we observed that the ratio between both isoforms (LC3-II/LC3-I) increased in CCCP-treated cells in a time-dependent manner, peaking at 12 h treatment (Figs. 1F and G). To affirm that autophagic flux is active, we measured LC3 turnover by lysosomes, using the mCherry-GFP-LC3B plasmid (GFP fluorescence is quenched by the lower pH levels in autophagolysosomes, whereas mCherry fluorescence is not). The presence of the uncoupler increased the number of red puncta (mCherry-LC3B) and, therefore, the autophagolysosomes over time (21.0 ± 3.1% and 80.3 ± 10.0% at 3 and 24 h of CCCP treatment, respectively), whereas GFP did not accumulate (Figs. 1H and I). This confirms that the increase in LC3-II protein levels seen after CCCP treatment (Figs. 1F and G) was due to increased autophagy flux.

### Cytotoxic effect of CCCP

**Fig. 2:**
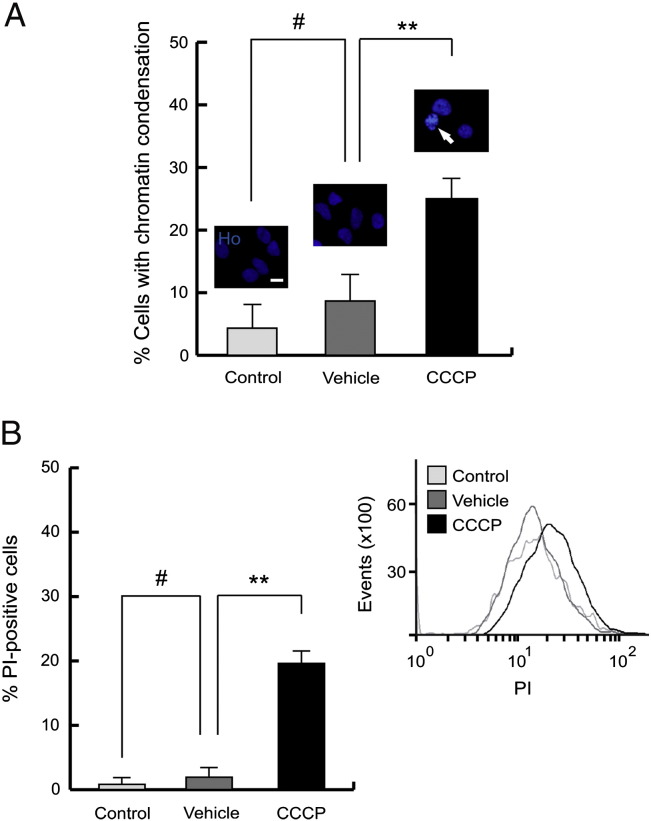
Cytotoxic effect of CCCP. (A) SH-SY5Y cells were exposed with 10 μM CCCP, with vehicle (0.05% (v/v) ethanol) or without any treatment (control) for 24 h, and labeled with Ho to measure the chromatin condensation. Graphic shows percentages of cells with chromatin condensation (#p > 0.05; **p ≤ 0.01). The arrows highlight nuclei with chromatin condensation. (B) SH-SY5Y cells were exposed with 10 μM CCCP, with vehicle (0.05% (v/v) ethanol) or without any treatment (control) for 24 h, harvested by trypsinization and labeled with PI to measure cell viability by flow cytometry. Percentages of PI-positive cells (#p > 0.05; **p ≤ 0.01) and representative single-parameter histogram of PI measurement. Data were expressed as mean ± SEM; events = 10,000.

To evaluate if this mitochondrial uncoupler could induce cell death, we measured typical patterns such as chromatin condensation and nuclear membrane permeabilization. Nuclei of untreated and vehicle-treated cells had no morphologic changes, while CCCP exposure increased the number of nuclei with chromatin condensation (24.9 ± 3.0%) (Fig. 2A) and positive PI staining (19.3 ± 4.5%) 24 h after treatment (Fig. 2B).

### CCCP effect on PINK1 protein levels

**Fig. 3:**
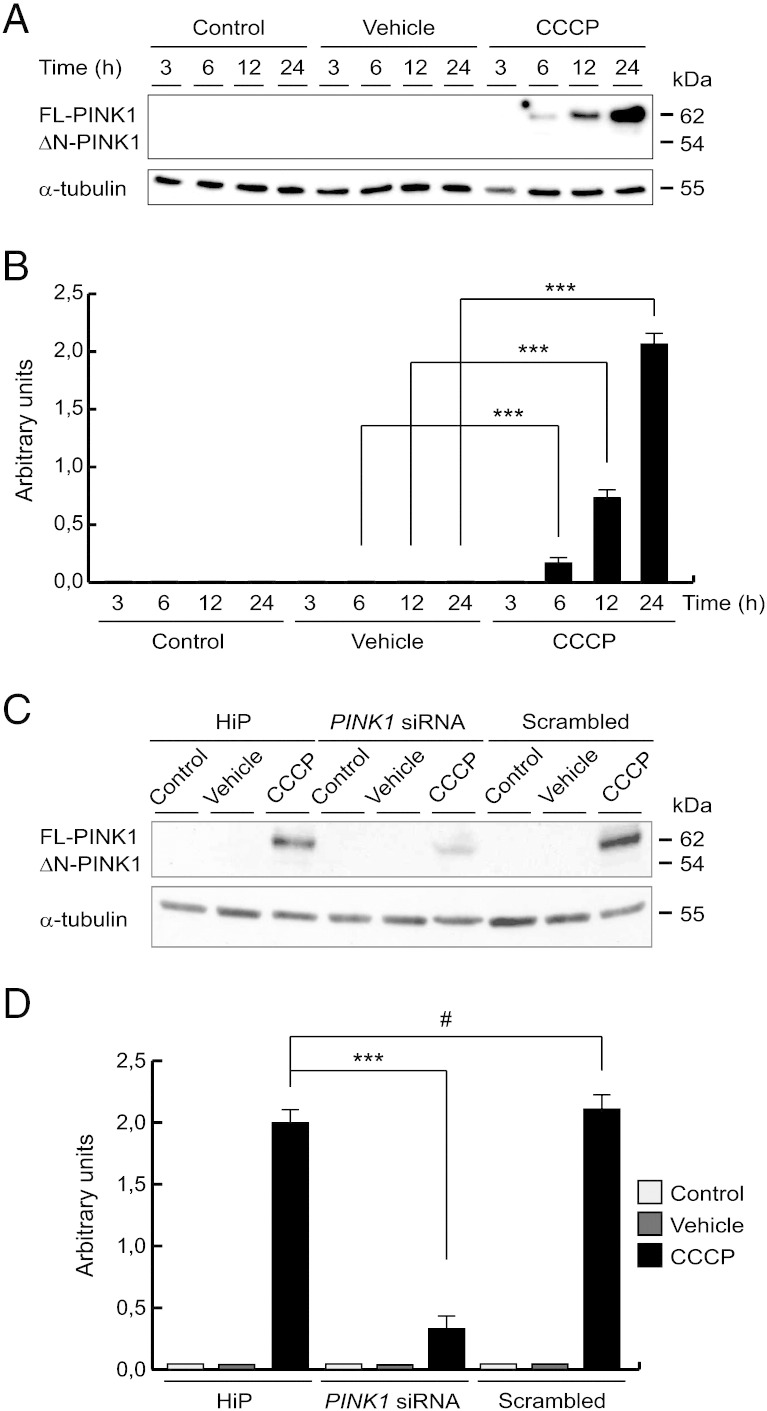
CCCP effect on PINK1 protein levels over time. (A and B) SH-SY5Y cells were exposed with 10 μM CCCP, with vehicle (0.05% (v/v) ethanol) or without any treatment (control), harvested by trypsinization at different times and lysed. The protein levels of PINK1 were determined by Western-blotting. α-tubulin expression was used as a loading control. (A) Representative blot of at least three independent experiments. (B) Densitometry of each band expressed in arbitrary units of intensity (***p ≤ 0.001). (C and D) SH-SY5Y cells were transfected with *PINK1* siRNA or scrambled control siRNA for 3 days and treated with 10 μM CCCP, with vehicle (0.05% (v/v) ethanol) or without any treatment (control), harvested by trypsinization at 24 h of treatment. The protein levels of PINK1 were determined by Western-blotting. α-tubulin expression was used as a loading control. (C) Representative blot of at least three independent experiments. (D) Densitometry of each band expressed in arbitrary units of intensity (#p > 0.05; ***p ≤ 0.001). Molecular mass is indicated in kDa next to the blots. Data were expressed as mean ± SEM; n = 3.

To further investigate the role of PINK1 in the mitochondrial damage, we determined the levels of PINK1 protein after CCCP exposure, demonstrating how the presence of the uncoupler produced a gradual and significant accumulation of endogenous FL-PINK1 protein in a time-dependent manner (Figs. 3A and B). Noticeably, the significant increase in FL-PINK1 after 24 h of CCCP treatment occurred at a time when mitochondrial content was decreased (Figs. 1A–C). Proteolytically processed forms of PINK1 (ΔN-PINK1) were undetectable in CCCP-treated cells. FL-PINK1 was not observed in untreated or vehicle-treated cells. However, FL-PINK1 levels fell within 15 min of CCCP washout, suggesting that its presence depends on the mitochondrial depolarization (data not shown). To confirm that the band detected was FL-PINK1, we silenced *PINK1* expression (Figs. 3C and D) or over-expressed full-length wild-type *PINK1* without a tag (data not shown).

### CCCP-induced mitophagy is due to mitochondrial PINK1 localization

**Fig. 4:**
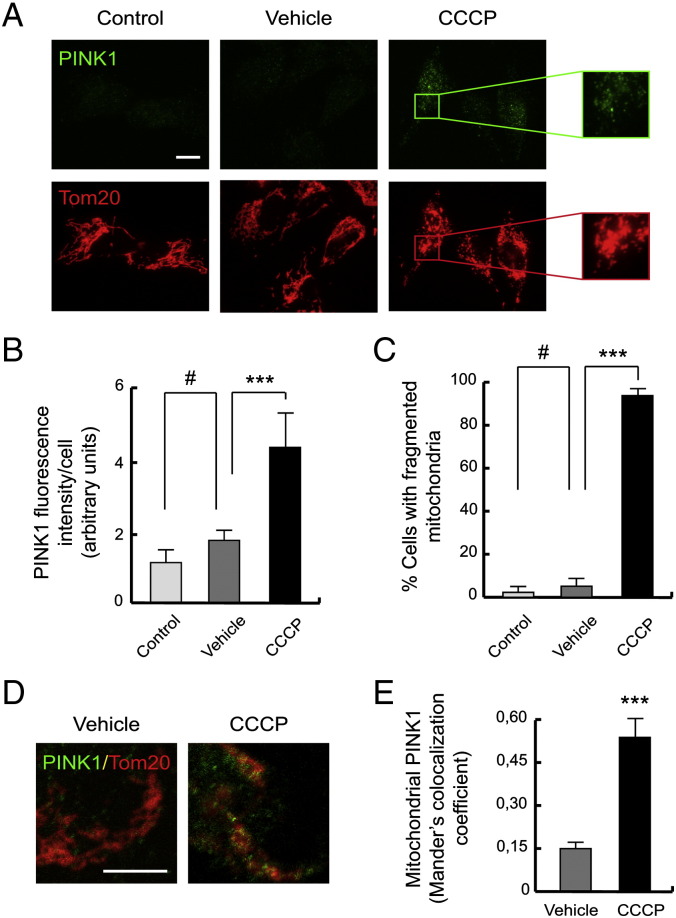
Mitochondrial localization of PINK1 in CCCP-treated SH-SY5Y cells. (A–C) SH-SY5Y cells were exposed 24 h with 10 μM CCCP, with vehicle (0.05% (v/v) ethanol) or without any treatment (control), fixed and immunostained for PINK1 (green) and Tom20 (red). (A) Representative immunofluorescence microphotographs. The boxes highlight high intensity of PINK1 and fragmented mitochondria, respectively. (B) Fluorescence intensity per cell (in AU), staining with anti-PINK1 antibody (#p > 0.05; ***p ≤ 0.001). (C) Percentages of cells with fragmented mitochondria, labeled with anti-Tom20 antibody (#p > 0.05; ***p ≤ 0.001). Scale bar represents 10 μm. Data were expressed as mean ± SEM; n = 200. (D and E) SH-SY5Y cells were immunostained after the treatment and performed with confocal techniques. (D) Representative confocal microphotographs, showing the localization of PINK1 (green) on mitochondria (Tom20; red). (E) Confocal analysis of the mitochondrial localization of PINK1, using Mander's colocalization coefficient (***p ≤ 0.001). Scale bar represents 10 μm.

We next examined the subcellular localization of PINK1, analyzing by immunofluorescence endogenous PINK1 and co-staining mitochondria with Tom20, an outer mitochondrial membrane protein. In control and vehicle-treated cells for 24 h, endogenous PINK1 levels were low (1.2 ± 0.4 AU of PINK1 fluorescence intensity/cell in control and 1.8 ± 0.3 in vehicle-treated cells) and mitochondria showed the typical tubular structure (3.2 ± 2.9% of cells with fragmented mitochondria in control and 5.9 ± 4.3% in vehicle-treated cells). Meanwhile, in CCCP-treated cells, levels of PINK1 increased (4.4 ± 1.0 AU of fluorescence intensity/cell) (Figs. 4A and B) and the mitochondrial network was fragmented (92.1 ± 4.8%) (Figs. 4A and C), showing how there is an accumulation of PINK1 in these cells co-localized with damaged mitochondria (Figs. 4D and E) (0.54 ± 0.09 Mander's colocalization coefficient in CCCP-treated cells compared to 0.15 ± 0.05 in vehicle-treated cells).

**Fig. 5:**
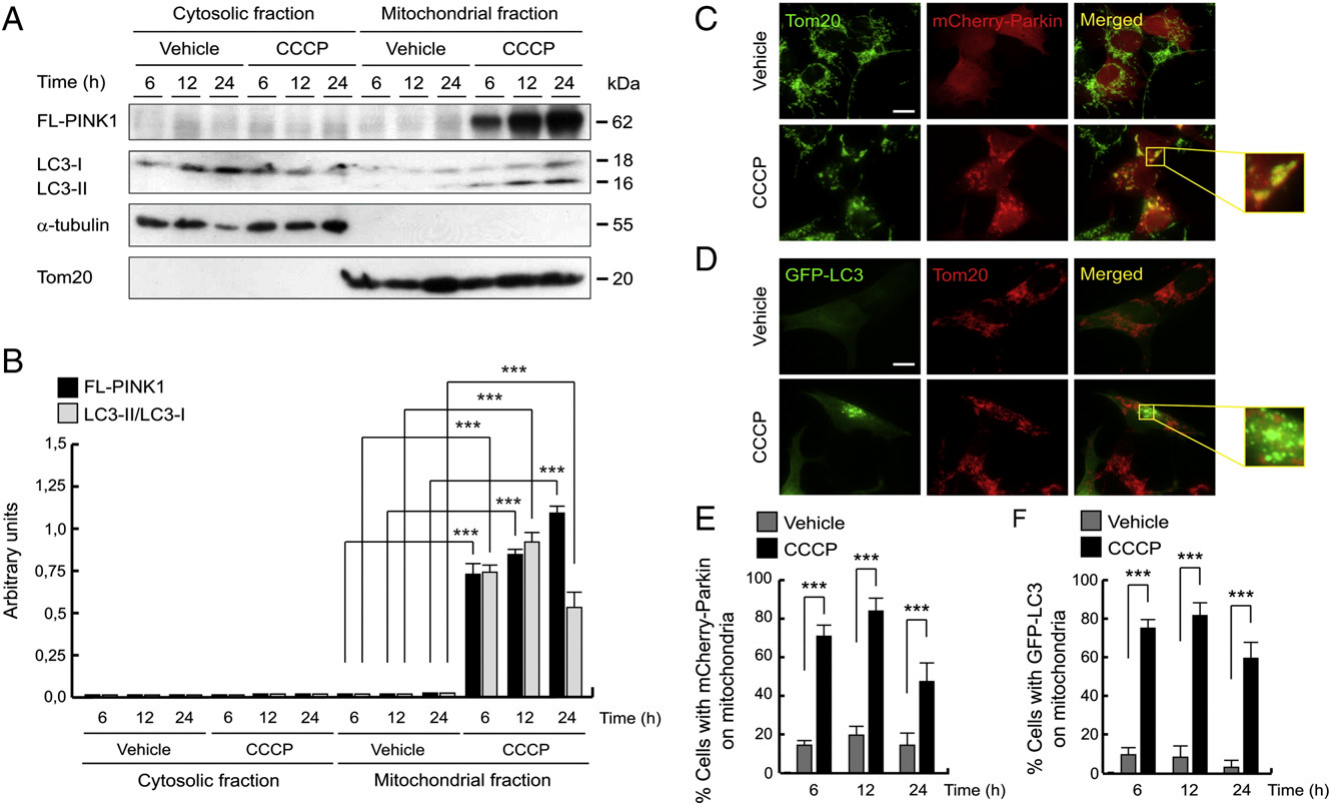
CCCP-induced mitophagy is parallel to mitochondrial PINK1 localization. (A and B) SH-SY5Y cells were exposed with 10 μM CCCP or with vehicle (0.05% (v/v) ethanol), harvested by trypsinization at different times, mitochondria isolated and Western-blotting performed. Blots were probed with antibodies against PINK1 and LC3B. α-tubulin and Tom20 were used as a cytosolic and mitochondrial loading control, respectively. (A) Representative blot of at least three independent experiments. (B) Densitometry of each band expressed in arbitrary units of intensity (***p ≤ 0.001). Molecular mass is indicated in kDa next to the blots. (C–F) SH-SY5Y cells were transfected with mCherry-Parkin or GFP-LC3 and exposed 6, 12 or 24 h with 10 μM CCCP or with vehicle (0.05% (v/v) ethanol), fixed and immunostained for Tom20 (green/red). (C and D) Representative immunofluorescence microphotographs of 6 hour treated-cells. The boxes highlight mitochondrial localization of mCherry-Parkin and GFP-LC3, respectively. (E) Percentages of cells with mCherry-Parkin on mitochondria, labeled with anti-Tom20 antibody (***p ≤ 0.001). (F) Percentages of cells with GFP-LC3 on mitochondria, labeled with anti-Tom20 antibody (***p ≤ 0.001). Scale bar represents 10 μm.

Furthermore, we observed that PINK1 levels increased in the mitochondria-rich fraction in a time-dependent manner after CCCP treatment, whereas in the cytosolic-rich fraction no band was observed (Figs. 5A and B). We also noted that there was an increase in LC3-II levels in the mitochondria rich fraction following CCCP treatment, being higher at 12 h (Figs. 5A and B). We confirmed this mitochondrial localization of LC3 by immunofluorescence, using GFP-LC3 plasmid (Figs. 5D and F). As with previous reports, accumulation of Parkin on mitochondria following CCCP treatment occurred. However, while PINK1 continued to accumulate on damaged mitochondria after 24 h, the amount of Parkin did not follow the same pattern (Figs. 5C and E), decreasing at this time, as LC3 protein (Figs. 5A, B, D and F).

### Increased *PINK1* gene expression after the CCCP exposure

**Fig. 6:**
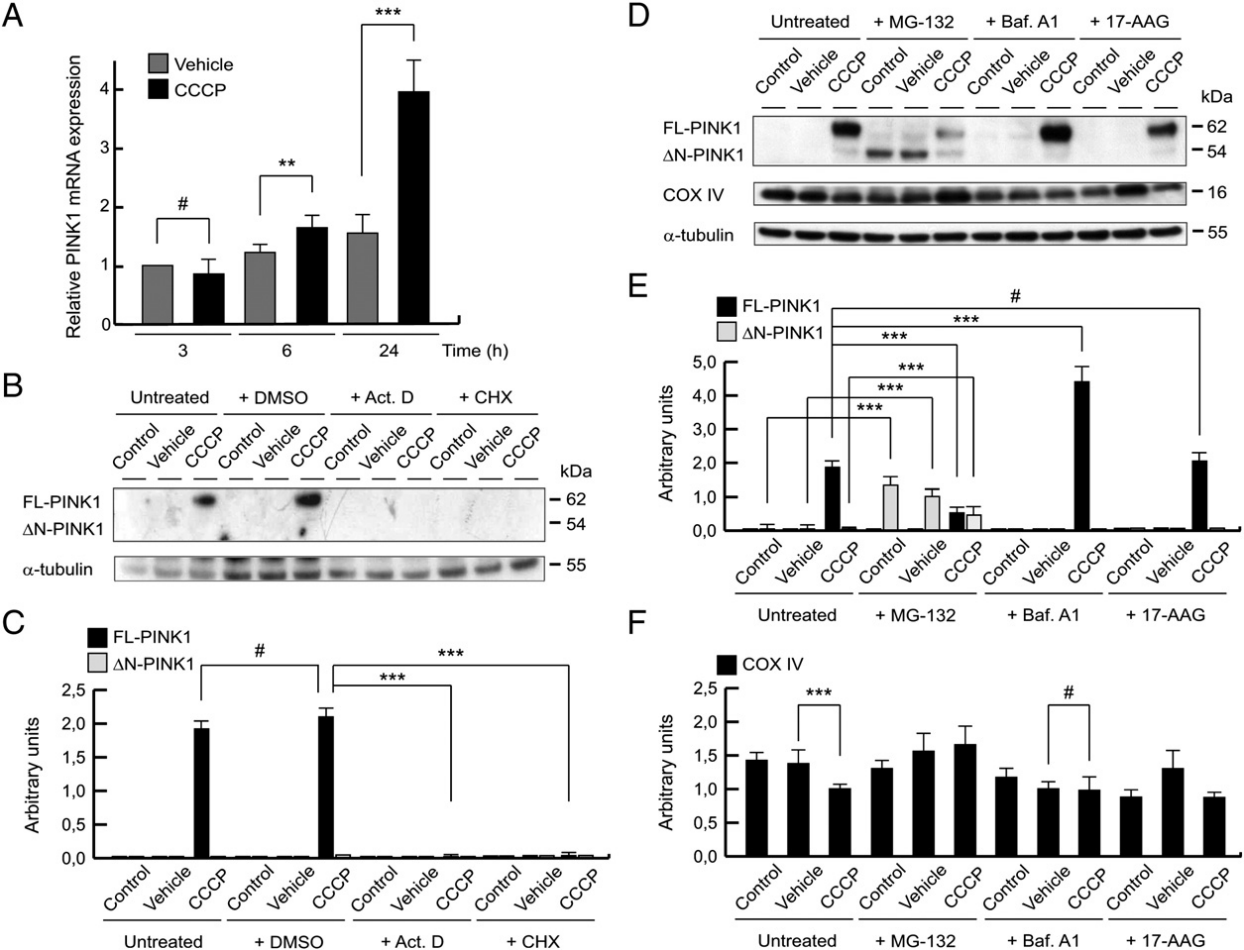
Increase of *PINK1* gene expression, and not its stabilization with chaperones, after the CCCP exposure. (A) SH-SY5Y cells were exposed with 10 μM CCCP or with vehicle (0.05% (v/v) ethanol) at different times, and PINK1 mRNA levels measured by reverse transcription and quantitative PCR. Relative expression was determined using GAPDH as housekeeping gene (#p > 0.05; **p ≤ 0.01; ***p ≤ 0.001). (B and C) SH-SY5Y cells were preincubated 1 h with 5 μg/ml Act. D, 100 μg/ml CHX or vehicle (0.1% (v/v) DMSO), exposed with 10 μM CCCP, with vehicle (0.05% (v/v) ethanol) or without any treatment (control) for 24 h, harvested by trypsinization and lysed. The protein levels of PINK1 were determined by Western-blotting. α-tubulin expression was used as a loading control. (B) Representative blot of at least three independent experiments. (C) Densitometry of each band expressed in arbitrary units of intensity (#p > 0.05; ***p ≤ 0.001). (D–F) SH-SY5Y cells were preincubated 1 h with 5 μM MG-132 or 100 nM Baf. A1, exposed with 10 μM CCCP, with vehicle (0.05% (v/v) ethanol) or without any treatment (control) for 24 h, incubated with 1 μM 17-AAG 3 h before collecting cells, harvested by trypsinization and lysed. The protein levels of PINK1 and COX IV were determined by Western-blotting. α-tubulin expression was used as a loading control. (D) Representative blot of at least three independent experiments. (E) Densitometry of each band expressed in arbitrary units of intensity (#p > 0.05; ***p ≤ 0.001). (F) Densitometry of each band expressed in arbitrary units of intensity (#p > 0.05; ***p ≤ 0.001). Molecular mass is indicated in kDa next to the blots. Data were expressed as mean ± SEM; n = 3.

We assessed whether the increase of endogenous PINK1 in mitochondria after CCCP treatment was due to an increase in gene expression level or a possible chaperone-mediated stabilization. For this purpose, we measured the mRNA levels of PINK1 by qPCR. In a short period of time (3 h), there were no differences, but we observed a significant increase of the PINK1 mRNA in a time-dependent manner, and was significantly higher at 6 (1.76 ± 0.22 times more than vehicle-treated cells at 3 h) and 24 h after CCCP treatment (4.01 ± 0.66 times more than vehicle-treated cells at 3 h) (Fig. 6A). These results were consistent when we studied the blockade of transcription and translation, using Act. D and CHX, respectively. The treatment with both inhibitors abolished expression of endogenous FL-PINK1 protein (Figs. 6B and C).

**Fig. S1:**
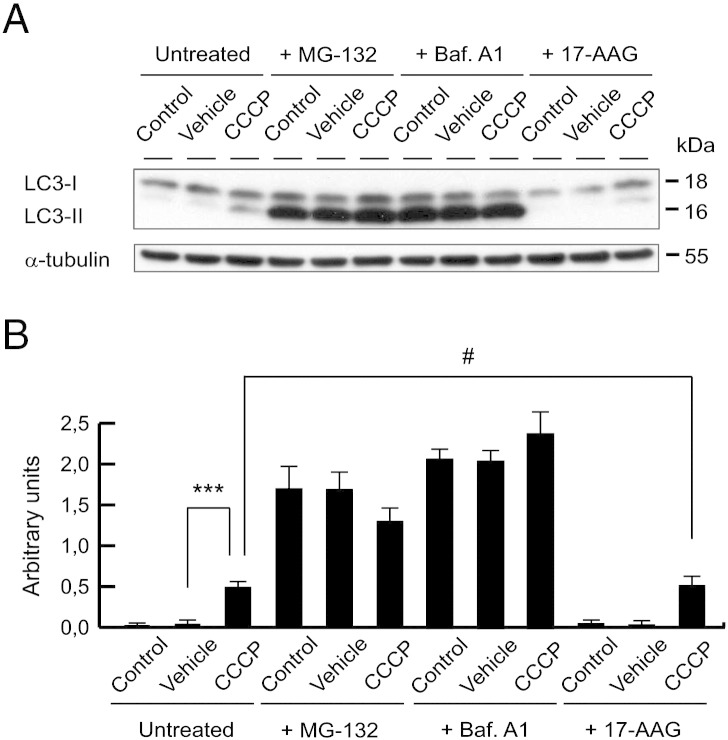
LC3 levels after UPS/autophagy blockade or 17-AAG treatment. (A and B) SH-SY5Y cells were preincubated 1 h with 5 μM MG-132 or 100 nM Baf. A1, exposed with 10 μM CCCP, with vehicle (0.05% (v/v) ethanol) or without any treatment (control) for 24 h, incubated with 1 μM 17-AAG 3 h before collecting cells, harvested by trypsinization and lysed. The LC3-II/LC3-I ratio was determined by Western-blotting. α-tubulin expression was used as a loading control. (A) Representative blot of at least three independent experiments. (B) Densitometry of each band expressed in arbitrary units of intensity (#p > 0.05; ***p ≤ 0.001). Molecular mass is indicated in kDa next to the blots. Data were expressed as mean ± SEM; n = 3.

Also, we investigated if degradation mechanisms, such as ubiquitin-proteasome system (UPS) and autophagy, modified PINK1 protein levels using MG-132 and bafilomycin A1 (Baf. A1), respectively. In this sense, there was an accumulation of ΔN-PINK1, but not FL-PINK1, in untreated and vehicle-treated cells after the treatment of MG-132, as previously reported (Lin and Kang, 2008, Takatori et al., 2008). Following CCCP treatment, ΔN-PINK1 levels were decreased, which was expected as FL-PINK1 accumulates on the outer mitochondrial membrane, thus reducing the amount of PINK1 available for cleavage by proteases in the inner mitochondrial membrane. In contrast, the levels of FL-PINK1 were further increased when we treated cells with CCCP and inhibited the autophagy mechanism with Baf. A1 (blocking autophagosome/lysosome fusion) (Figs. 6D and E). Western blotting for COX IV and LC3-II protein levels indicated that Baf. A1 prevented the CCCP-induced decrease in mitochondrial content (Figs. 6D and F) and inhibited autophagy flux (Fig. S1). This suggests that the further increase in FL-PINK1 levels after CCCP and Baf. A1 treatment was most likely due to a greater number of mitochondria on which PINK1 could accumulate. Finally, FL-PINK1 protein was still increased by CCCP in the presence of 17-AAG, an inhibitor of Hsp90 (Figs. 6D and E), further suggesting that the increase of FL-PINK1 at 24 h was due to de novo synthesis, rather than increased stabilization of PINK1 (Lin and Kang, 2008).

### Involvement of extracellular calcium in PINK1 levels

**Fig. 7:**
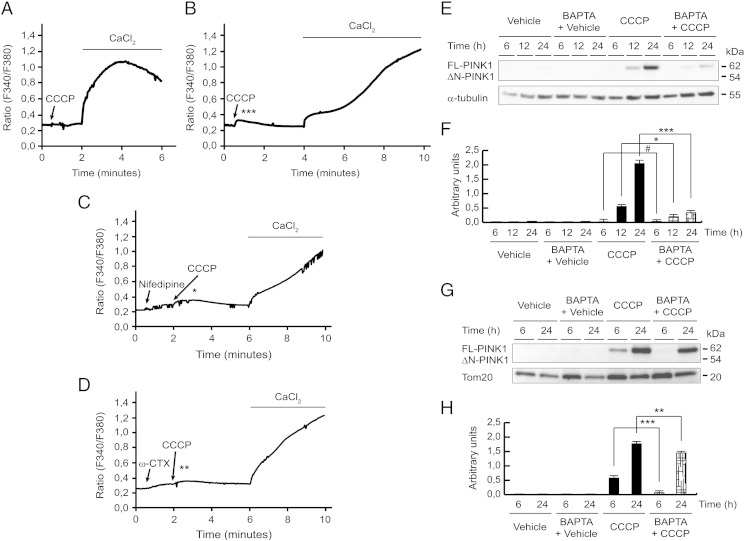
Involvement of extracellular calcium in PINK1 levels. (A–D) Time courses of Ratio (F340/F380) in Fura-2 AM loaded SH-SY5Y cells to determine cytosolic calcium changes. (A) Time course of [Ca^2 +^]_cyt_ changes in SH-SY5Y cells after the addition of 10 μM CCCP in Ca^2 +^-free Locke's K25 buffer. (B) Time course of [Ca^2 +^]_cyt_ changes in SH-SY5Y cells induced by 10 μM CCCP in complete Locke's K25 buffer (***p ≤ 0.001 between + Ca^2 +^-CCCP-treated (n = 22) and − Ca^2 +^-CCCP-treated cells (n = 12)). (C) Time course of [Ca^2 +^]_cyt_ changes in SH-SY5Y cells after the treatment of 10 μM nifedipine and 10 μM CCCP in complete Locke's K25 buffer (*p ≤ 0.05 between + Ca^2 +^-nifedipine-CCCP-treated (n = 10) and + Ca^2 +^-CCCP-treated cells). (D) Time course of [Ca^2 +^]_cyt_ changes in SH-SY5Y cells in the presence of 2 μM ω-CTX and 10 μM CCCP in complete Locke's K25 buffer (**p ≤ 0.01 between + Ca^2 +^-ω-CTX-CCCP-treated (n = 31) and + Ca^2 +^-CCCP-treated cells). (E and F) SH-SY5Y cells were preincubated 1 h with 5 μM BAPTA-AM, exposed with 10 μM CCCP or with vehicle (0.05% (v/v) ethanol), harvested by trypsinization at different times and lysed. The protein levels of PINK1 were determined by Western-blotting. α-tubulin expression was used as a loading control. (E) Representative blot of at least three independent experiments. (F) Densitometry of each band expressed in arbitrary units of intensity (#p > 0.05; *p ≤ 0.05; ***p ≤ 0.001). (G and H) SH-SY5Y cells were preincubated 1 h with 5 μM BAPTA-AM, exposed 6 or 24 h with 10 μM CCCP or with vehicle (0.05% (v/v) ethanol), mitochondrial isolated and Western-blotting performed. Tom20 was used as a mitochondrial loading control. (G) Representative blot of at least three independent experiments. (H) Densitometry of each band expressed in arbitrary units of intensity (**p ≤ 0.01; ***p ≤ 0.001). Molecular mass is indicated in kDa next to the blots. Data were expressed as mean ± SEM; n = 3.

To examine whether the treatment with CCCP stimulated calcium signaling and if this is associated with increased transcription of PINK1, leading to the accumulation of FL-PINK1 on mitochondria, we measured intracellular calcium mobilization in SH-SY5Y cells using the ratiometric indicator Fura-2 AM. We found that the presence of CCCP did not modify the release of calcium from an intracellular store (Fig. 7A), but produced the entry of extracellular calcium and consequently an elevation in cytosolic calcium levels immediately after treatment (Fig. 7B). Next, we evaluated the mechanism of extracellular calcium entry. We investigated whether calcium entry was dependent on L- and N-type VDCCs, which are well characterized in SH-SY5Y cells (Zhang et al., 2009) and have been implicated in PD pathogenesis (Goldberg et al., 2012, Ilijic et al., 2011). We used 10 μM nifedipine and 2 μM ω-CTX, and showed that the treatment with these VDCC blockers significantly reduced extracellular calcium entry (Figs. 7C and D). Moreover, the blockade of intracellular calcium release from stores such as ER (using thapsigargin), mitochondria (with rotenone) and lysosomes (using Baf. A1) did not prevent the initial increase in intracellular calcium observed after treatment with CCCP (data not shown).

**Fig. S2:**
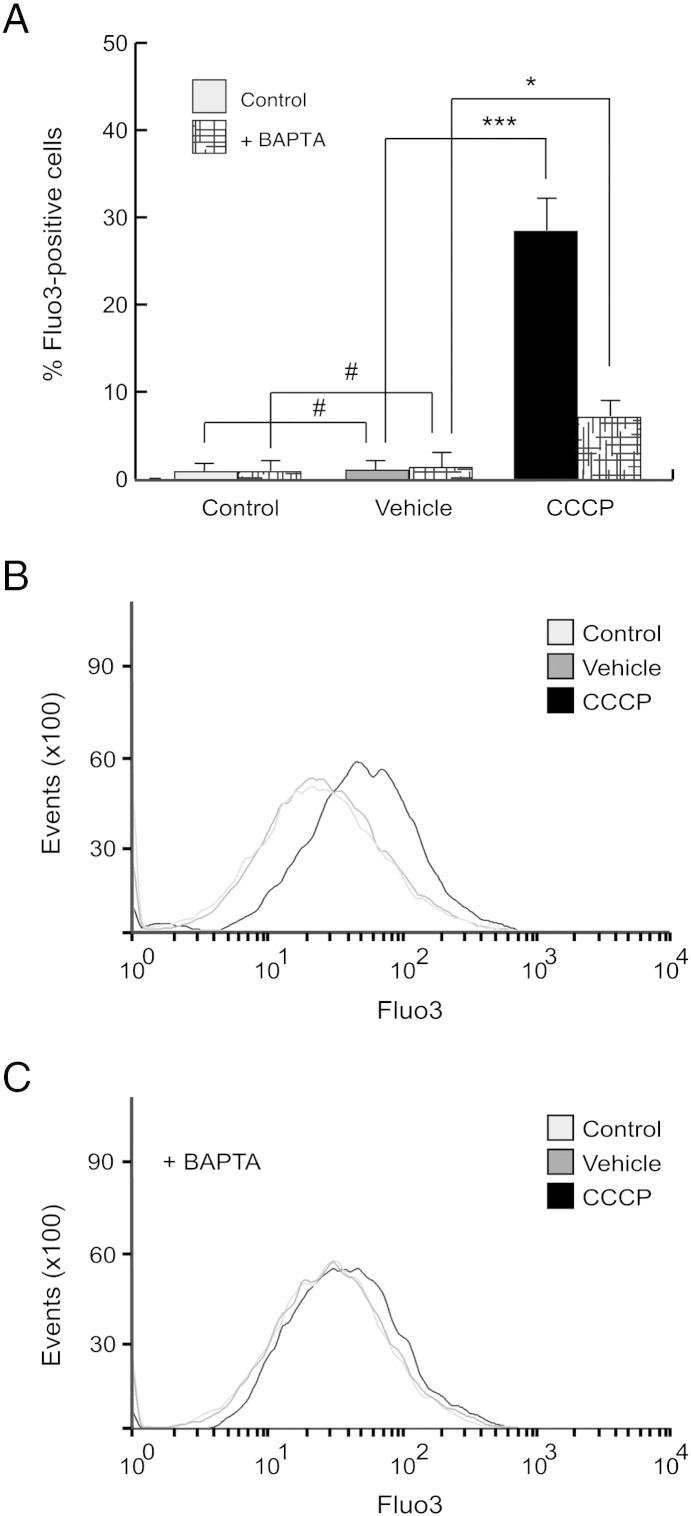
Calcium chelation by BAPTA-AM. SH-SY5Y cells were preincubated 1 h with 5 μM BAPTA-AM, exposed with 10 μM CCCP or with the vehicle (0.05% (v/v) ethanol) or without any treatment until 24 h, harvested by trypsinization and labeled with Fluo3 to measure cytosolic calcium by flow cytometry. (A) Percentages of Fluo3-positive cells (#p > 0.05; *p ≤ 0.05; ***p ≤ 0.001). (B and C) Representative single-parameter histograms of Fluo3 measurement. Data were expressed as mean ± SEM; events = 10,000.

**Fig. S3:**
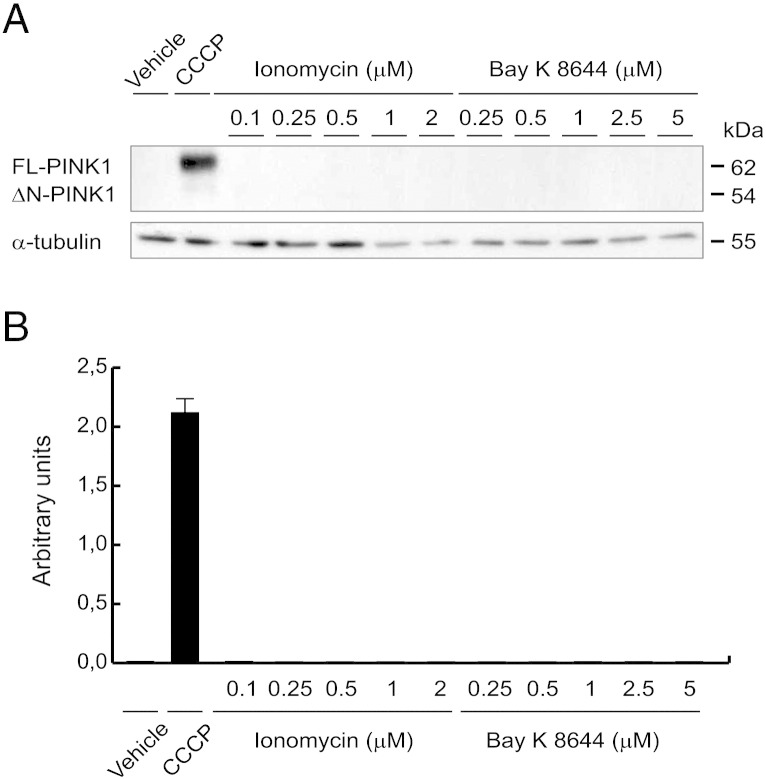
PINK1 protein levels after ionomycin or Bay K 8644 treatment. (A and B) SH-SY5Y cells were exposed with 10 μM CCCP, with the vehicle (0.05% (v/v) ethanol), with ionomycin or Bay K 8644 for 24 h, lysates prepared and Western-blotting performed. Blots were probed with antibodies against *PINK1*. α-tubulin was used as a loading control. (A) Representative blot of at least three independent experiments. (B) Densitometry of each band expressed in arbitrary units of intensity. Molecular mass is indicated in kD next to the blots. Data were expressed as mean ± SEM; n = 3.

Next, we analyzed the role of this intracellular calcium elevation after the CCCP treatment on the PINK1 levels and its possible importance in the PINK1 mitochondrial localization. We observed that calcium chelation with BAPTA-AM decreased FL-PINK1 levels after CCCP treatment, both in cell lysates (Figs. 7E and F) and isolated mitochondria (Figs. 7G and H). Chelation of calcium by BAPTA-AM was confirmed by flow cytometry (Fig. S2). Furthermore, to assess whether cytosolic calcium increase could mimic the CCCP effects on PINK1 protein, cells were exposed to the calcium ionophore ionomycin and the L-type VDCC agonist Bay K 8644, showing that both did not modify FL-PINK1 protein levels, comparable to vehicle-treated cells (Fig. S3).

### *PINK1* calcium-dependent gene expression

**Fig. 8:**
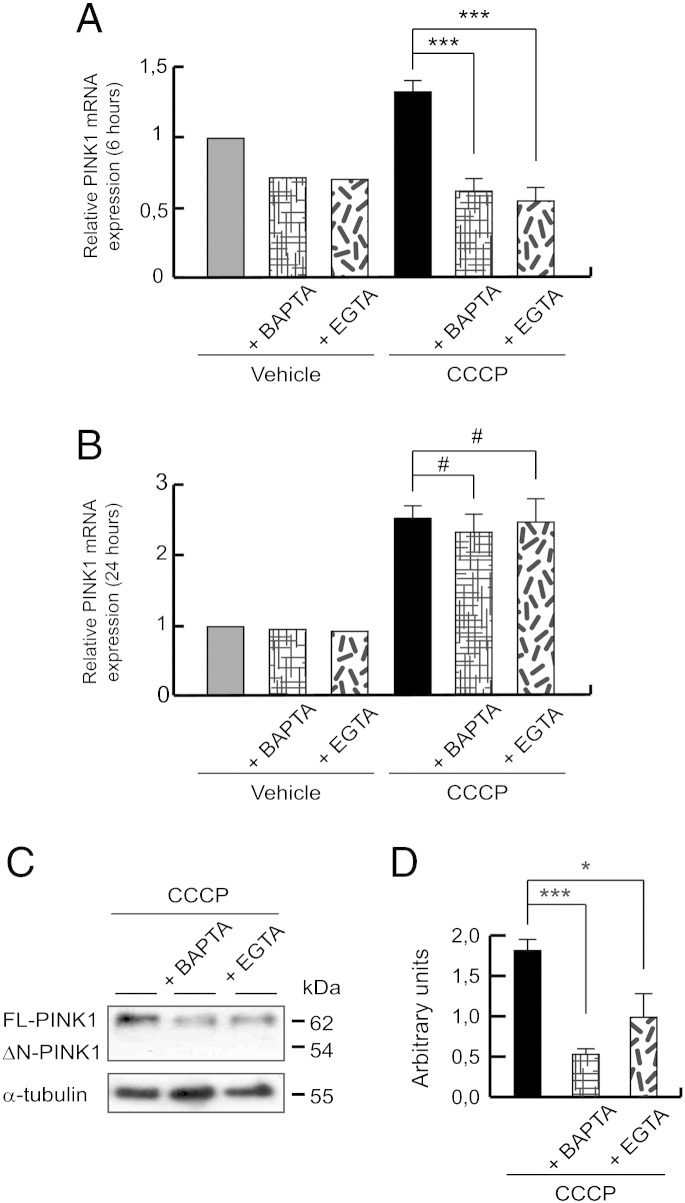
*PINK1* calcium-dependent gene expression. (A and B) SH-SY5Y cells were preincubated 1 h with 5 μM BAPTA-AM or 500 μM EGTA, exposed 6 or 24 h with 10 μM CCCP or with vehicle (0.05% (v/v) ethanol), and PINK1 mRNA levels measured by reverse transcription and quantitative PCR. Relative expression was determined using GAPDH as housekeeping gene antibody. (A) Relative PINK1 mRNA expression after 6 hour CCCP exposure and calcium chelation (***p ≤ 0.001). (B) Relative PINK1 mRNA expression after 24 hours of CCCP treatment and calcium chelation (#p > 0.05). (C and D) SH-SY5Y cells were preincubated 1 h with 5 μM BAPTA-AM or 500 μM EGTA, exposed 24 h with 10 μM CCCP, harvested by trypsinization and lysed. The protein levels of PINK1 were determined by Western-blotting. α-tubulin expression was used as a loading control. (C) Representative blot of at least three independent experiments. (D) Densitometry of each band expressed in arbitrary units of intensity (*p ≤ 0.05; ***p ≤ 0.001). Molecular mass is indicated in kDa next to the blots. Data were expressed as mean ± SEM; n = 3.

To elucidate if extracellular calcium increases the PINK1 mitochondrial localization by enhancing its gene expression, PINK1 mRNA levels were measured by qPCR. PINK1 mRNA levels were significantly decreased after 6 h of CCCP treatment when cytosolic calcium was eliminated by BAPTA-AM (0.69 ± 0.09 times less than 6 hour CCCP-treated cells) or extracellular calcium in the medium was chelated by EGTA (0.77 ± 0.10 times less than 6 hour CCCP-treated cells) (Fig. 8A). But, if the exposure is longer (24 h), these rates did not modulate when we sequestered cytosolic (0.18 ± 0.25 times less than CCCP-treated cells) and extracellular calcium (0.09 ± 0.62 times less than CCCP-treated cells) (Fig. 8B). However, it would appear that decreased transcription of PINK1 mRNA by BAPTA-AM or EGTA for at least 6 h was sufficient to decrease FL-PINK1 protein levels after 24 h of CCCP treatment (Figs. 7E–H, 8C and D).

### Nuclear recruitment and activation of c-Fos after the CCCP treatment

**Fig. 9:**
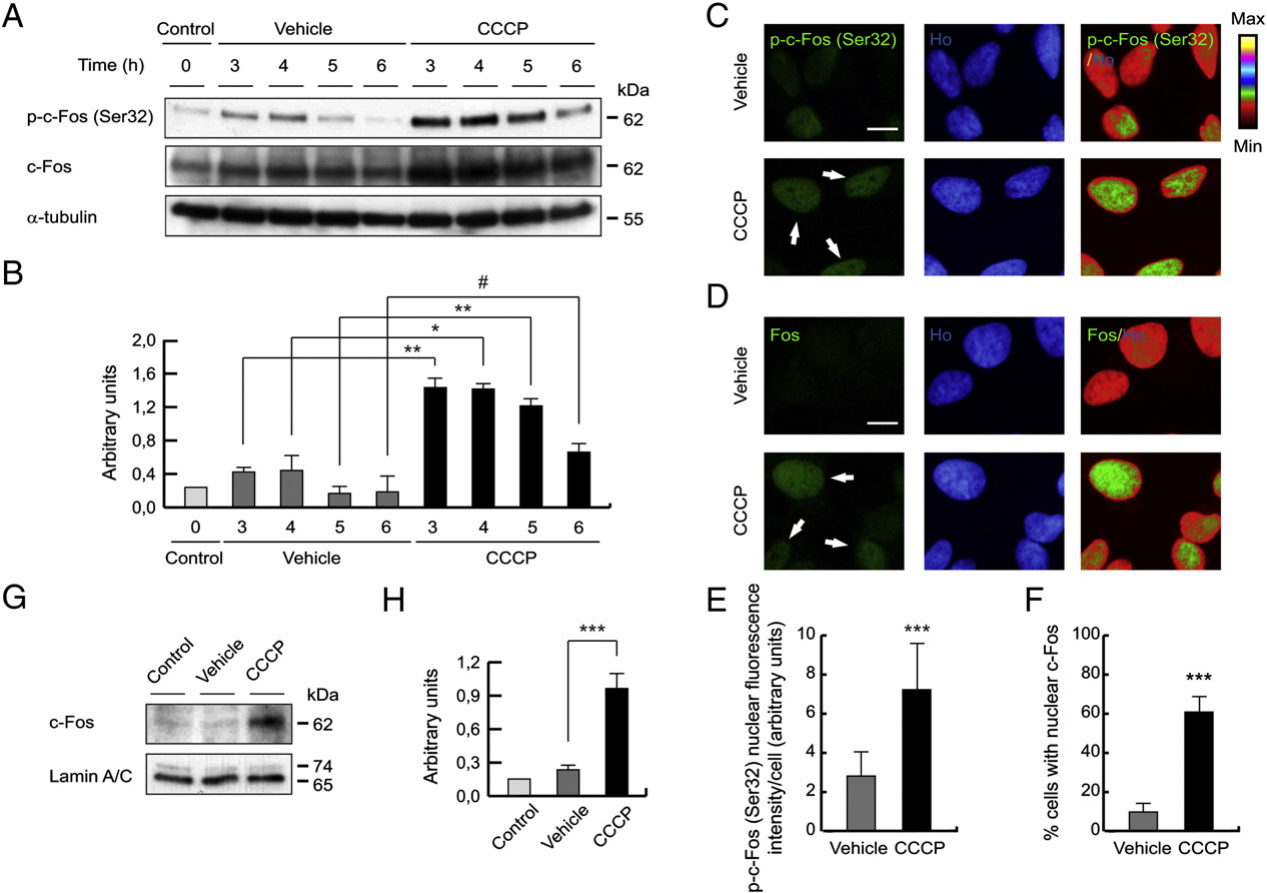
Nuclear recruitment of c-Fos after the CCCP treatment. (A and B) SH-SY5Y cells were exposed with 10 μM CCCP, with vehicle (0.05% (v/v) ethanol) or without any treatment (control), harvested by trypsinization at different times and lysed. The protein levels of p-c-Fos (Ser32) and c-Fos were determined by Western-blotting. α-tubulin expression was used as a loading control. (A) Representative blot of at least three independent experiments. (B) Densitometry of each band expressed in arbitrary units of intensity (#p > 0.05; *p ≤ 0.05; **p ≤ 0.01). (C–F) SH-SY5Y cells were exposed 3 h with 10 μM CCCP or with vehicle (0.05% (v/v) ethanol), fixed and immunostained for p-c-Fos (Ser32) or c-Fos (green) and Ho (blue). (C) Representative immunofluorescence microphotographs. The arrows highlight high nuclear intensity of p-c-Fos (Ser32). (D) Representative immunofluorescence microphotographs. The arrows highlight c-Fos nuclear staining. (E) Nuclear fluorescence intensity per cell (in AU), staining with anti-p-c-Fos (Ser32) antibody (***p ≤ 0.001). (F) Percentages of cells with nuclear c-Fos (***p ≤ 0.001). Scale bar represents 10 μm. Data were expressed as mean ± SEM; n = 200. (G and H) SH-SY5Y cells were exposed 3 h with 10 μM CCCP, with vehicle (0.05% (v/v) ethanol) or without any treatment (control), nuclear isolated and Western-blotting performed. Lamin A/C was used as a nuclear loading control. (G) Representative blot of at least three independent experiments. (H) Densitometry of each band expressed in arbitrary units of intensity (***p ≤ 0.001). Molecular mass is indicated in kDa next to the blots. Data were expressed as mean ± SEM; n = 3.

**Fig. S4:**
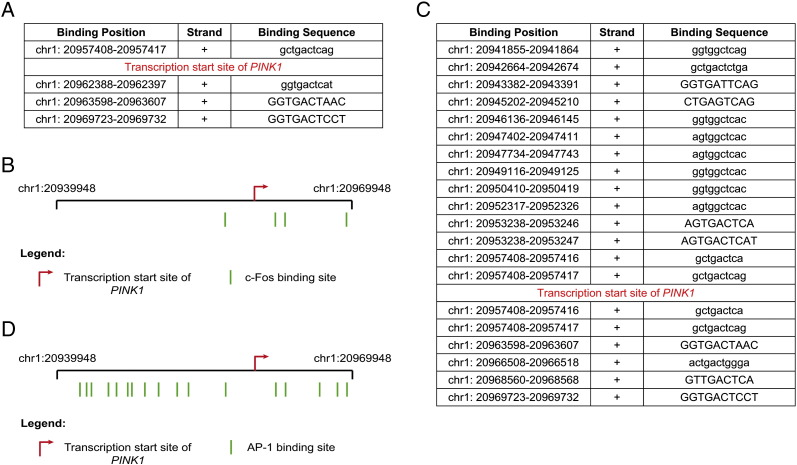
c-Fos and AP-1 response elements in the *PINK1* promoter. (A and B) c-Fos binding sites in the *PINK1* gene, showed in a detailed table (A) and in the gene map (B). (C and D) AP-1 binding sites in the *PINK1* gene, showed in a detailed table (C) and in the gene map (D). Modified from the sabiosciences webpage (http://www.sabiosciences.com/chipqpcrsearch.php?app=TFBS).

To further evaluate the role of calcium in *PINK1* expression, we performed some experiments to elucidate whether this higher gene expression under CCCP treatment is modulated by a transcription factor, following activation by calcium. Bioinformatic analysis of the PINK1 promoter indicated four c-Fos response elements (Fig. S4). We observed that CCCP treatment increased the phosphorylation of c-Fos at residue Ser32 between 3 and 6 h, when compared with cells treated with the vehicle (Figs. 9A and B). Immunofluorescence of c-Fos also showed that CCCP treatment produced higher levels of p-c-Fos (Ser32) (7.06 ± 2.85 AU of nuclear fluorescence intensity/cell), compared to vehicle-treated cells (2.84 ± 1.27 AU) (Figs. 9C and E). The nuclear localization of c-Fos was also increased by CCCP treatment (58.22 ± 11.02% of CCCP-treated cells vs. 11.36 ± 5.80% of vehicle-treated cells) (Figs. 9D and F). The nuclear translocation of c-Fos was also confirmed by Western-blotting (Figs. 9G and H). We analyzed other factors modulated by calcium, such as CREB, c-Jun, FOXO3a and NFκB, but obtained inconclusive results (data not shown).

### c-Fos calcium-dependent signaling acts in parallel to *PINK1* expression after CCCP exposure

**Fig. 10:**
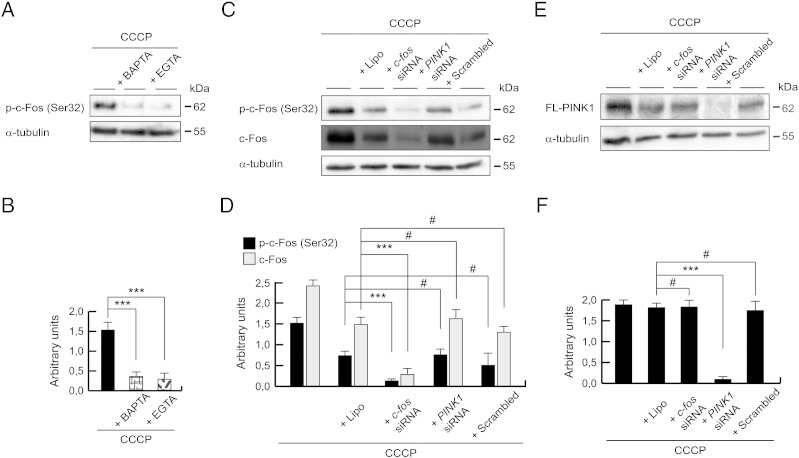
c-Fos-independent expression of *PINK1* after CCCP exposure. (A and B) SH-SY5Y cells were preincubated 1 h with 5 μM BAPTA-AM or 500 μM EGTA, exposed 3 h with 10 μM CCCP, harvested by trypsinization and lysed. The protein levels of p-c-Fos (Ser32) were determined by Western-blotting. α-tubulin expression was used as a loading control. (A) Representative blot of at least three independent experiments. (B) Densitometry of each band expressed in arbitrary units of intensity (***p ≤ 0.001). (C–F) SH-SY5Y cells were transfected with *c-fos* siRNA, *PINK1* siRNA or scrambled control siRNA for 2 days and treated with 10 μM CCCP, harvested by trypsinization at different times and lysed. The protein levels of p-c-Fos (Ser32), c-Fos and PINK1 were determined by Western-blotting. α-tubulin expression was used as a loading control. (C) Representative blot of at least three independent experiments, from 3 hour-CCCP-treated cells. (D) Densitometry of each band expressed in arbitrary units of intensity (#p > 0.05; ***p ≤ 0.001). (E) Representative blot of at least three independent experiments, from 24 hour-CCCP-treated cells. (F) Densitometry of each band expressed in arbitrary units of intensity (#p > 0.05; ***p ≤ 0.001). Molecular mass is indicated in kDa next to the blots. Data were expressed as mean ± SEM; n = 3.

To elucidate if extracellular calcium enhanced *PINK1* expression via c-Fos activation, firstly, we analyzed the phosphorylation of c-Fos after a combination of CCCP exposure and deprivation of cytosolic or extracellular calcium. Both BAPTA-AM and EGTA treatment with CCCP decreased phosphorylation of c-Fos (Figs. 10A and B). Finally, we set out to determine whether the c-Fos/PINK1 axis was in the same pathway. Following *c-fos* gene silencing, PINK1 protein levels did not change (Figs. 10E and F) after the 24 h CCCP treatment, and vice versa, the silencing of *PINK1* did not affect the c-Fos phosphorylation, that appeared when cells were exposed to uncoupler for 3 h (Figs. 10C and D). Further studies are needed to elucidate which mechanisms control *PINK1* gene expression.

### CCCP-induced mitophagy is calcium-dependent, but is a c-Fos independent mechanism

**Fig. 11:**
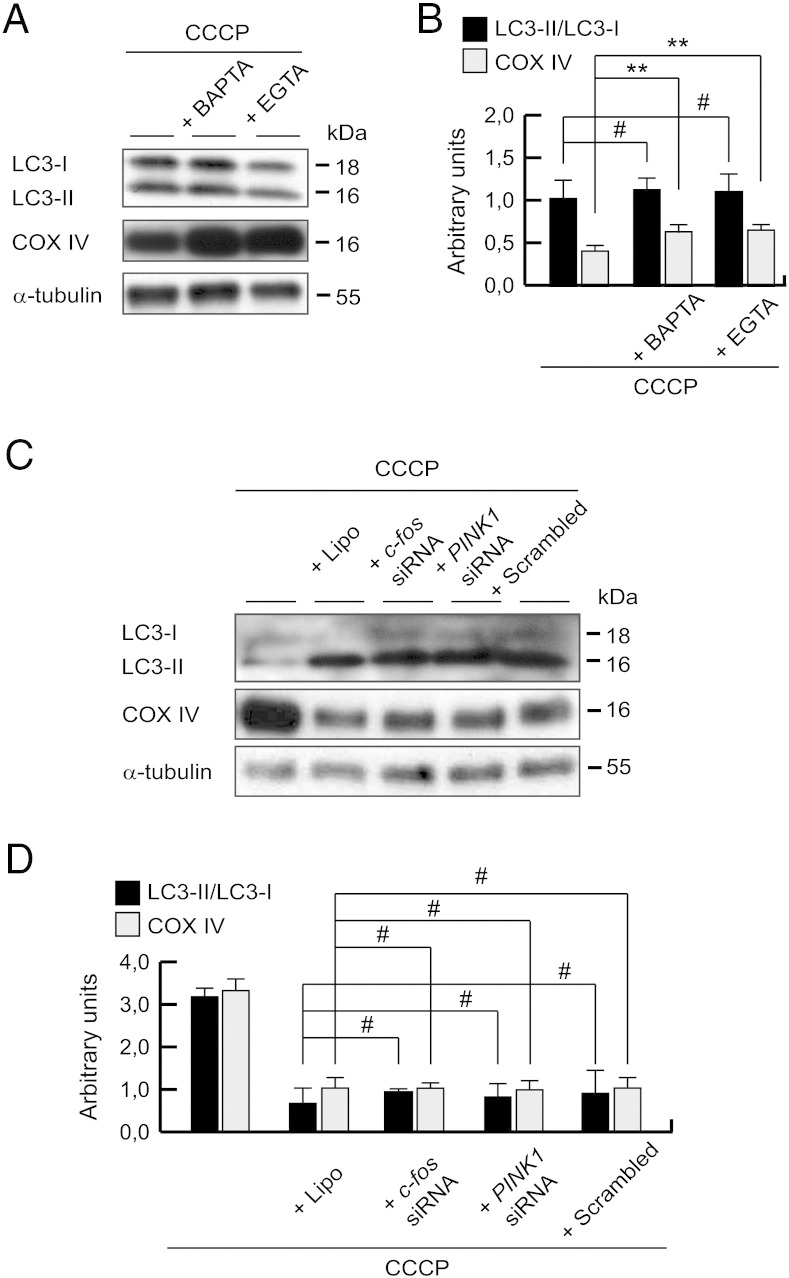
Mitophagy decreases after calcium chelation, but is a c-Fos-independent mechanism. (A and B) SH-SY5Y cells were preincubated 1 h with 5 μM BAPTA-AM or 500 μM EGTA, exposed 24 h with 10 μM CCCP, harvested by trypsinization and lysed. The ratio LC3-II/LC3-I and protein levels of COX IV were determined by Western-blotting. α-tubulin expression was used as a loading control. (A) Representative blot of at least three independent experiments. (B) Densitometry of each band expressed in arbitrary units of intensity (#p > 0.05; **p ≤ 0.01). (C and D) SH-SY5Y cells were transfected with *c-fos* siRNA, *PINK1* siRNA or scrambled control siRNA for 2 days and treated 24 h with 10 μM CCCP, harvested by trypsinization and lysed. The ratio LC3-II/LC3-I and protein levels of COX IV were determined by Western-blotting. α-tubulin expression was used as a loading control. (C) Representative blot of at least three independent experiments. (D) Densitometry of each band expressed in arbitrary units of intensity (#p > 0.05). Molecular mass is indicated in kDa next to the blots. Data were expressed as mean ± SEM; n = 3.

At this point, we considered whether mitophagy could be modulated by calcium and/or c-Fos signaling. SH-SY5Y cells treated with CCCP and BAPTA-AM or EGTA increased COX IV protein levels, when compared to cells treated with CCCP alone. However, LC3 protein levels were increased to a similar extent in cells treated with CCCP alone, or in combination with BAPTA-AM or EGTA (Figs. 11A and B). Moreover, if we analyzed LC3 and COX IV levels after the *c-fos* gene silencing, we observed the same pattern as the Lipofectamine condition (Figs. 11C and D), showing how this reagent generates an accumulation of LC3-II protein levels (Mo et al., 2012).

## Discussion

We report that depolarization of mitochondria with CCCP in SH-SY5Y cells results in a calcium-dependent increase in transcription of *PINK1*, and not just stabilization of PINK1 protein on the outer mitochondrial membrane. Previously, it has been shown that FL-PINK1 accumulates on the outer mitochondrial membrane of mitochondria within 15 min (Matsuda et al., 2010, Narendra et al., 2010b, Shiba-Fukushima et al., 2012). Similar to these previous reports, we also found that the accumulation of FL-PINK1 was diminished after 15 min of CCCP being removed, presumably because upon restoration of the mitochondrial membrane potential, FL-PINK1 was able to proceed to the inner mitochondrial membrane to be processed by mitochondrial proteases (Deas et al., 2011, Greene et al., 2012, Jin et al., 2010) and then be degraded by the proteasome (Jin et al., 2010, Lin and Kang, 2008, Takatori et al., 2008).

These reports have typically looked at FL-PINK1 protein levels up to 6 h after CCCP treatment. However, we are not aware of any reports of PINK1 mRNA levels following CCCP treatment. We found that PINK1 mRNA levels were significantly increased after 6 h of CCCP treatment and continued to rise for 24 h. This resulted in a continued increase in de novo FL-PINK1 protein, which accumulated on mitochondria. The increase in FL-PINK1 protein at the mitochondria after 24 h occurred despite a 29% decrease in mitochondrial content. This highlights the importance of PINK1 on damaged mitochondria. It should be noted that we found that washing away of CCCP after 6 h, and measuring mitochondrial content 18 h later (24 h in total), no longer resulted in a significant decrease in mitochondria content. This is despite the fact that the recruitment of Parkin to mitochondria by PINK1 (Matsuda et al., 2010, Narendra et al., 2010b, Vives-Bauza et al., 2010), and the PINK1/Parkin-dependent ubiquitination of mitochondrial proteins such as VDAC and the mitofusins, and the mitochondrial transport protein Miro, occurred within 3 h of mitophagy induction (Chan et al., 2011, Gegg et al., 2010, Geisler et al., 2010, Tanaka et al., 2010). Our results suggest that maximum activity of mitophagy (as measured by colocalization of LC3 with mitochondria) occurred at 12 h of CCCP treatment, meanwhile mitochondrial PINK1 localization continued increasing for at least 24 h. This suggests that FL-PINK1 might have further functions in mitochondrial quality control downstream of the recruitment of Parkin and ubiquitination of mitochondrial proteins.

Higher *PINK1* expression could be linked with a compensatory increase in mitochondrial biogenesis. The induction of mitophagy would require the cell to initiate synthesis of new mitochondria to replace them. PINK1 has been linked with mitochondrial biogenesis, with knockdown of PINK1 in SH-SY5Y cells decreasing mtDNA levels and mtDNA synthesis (Gegg et al., 2009). Induction of mitochondrial uncoupling proteins can induce mitochondrial biogenesis via the transcription factor Nrf1 (Li et al., 1999). Therefore it is unclear whether any putative increase in mitochondrial biogenesis following CCCP treatment is a direct response to mitochondrial uncoupling or the removal of mitochondria.

This increase in transcription appeared to be due to an increase in intracellular calcium levels. Treatment of SH-SY5Y cells with CCCP increased calcium levels within the cell, with uptake of calcium into cells via the L- and N-type VDCCs as a major component. In this sense, higher levels of intracellular calcium after FCCP (CCCP analog) exposure are reduced in calcium-free solution or by VDCC blockade (Buckler and Vaughan-Jones, 1998, Sato, 1997). More sensitive techniques are required to determine if small local increases in cytosolic calcium could also be caused as a result of increased mitochondrial release upon depolarization (Tonkikh and Carlen, 2009). The increase in PINK1 expression did not occur when intracellular calcium was increased by ionomycin or an L-VDCC agonist. Ionomycin treatment has previously been shown not to result in the phosphorylation of Ser65 of Parkin by PINK1, an early event in mitophagy (Kondapalli et al., 2012). We suspect that we did not see the increase in PINK1 transcription with these two treatments because the mitochondria were still healthy, and were thus still able to buffer the calcium overload generated by ionophores or the L-VDCC agonist. While treatment with calcium chelators prevented loss of mitochondrial content following 24 h of CCCP treatment, further work is required to determine whether this is directly due to the decrease in PINK1 transcription, or other effects of decreased calcium on other components of the autophagy machinery.

Therefore, the induction of *PINK1* expression might simply be a protective measure against the increased cytosolic calcium observed after CCCP treatment. Indeed, there was an increase in nuclear condensation and PI-positive cells following 24 h of CCCP treatment. PINK1 expression is known to protect against a variety of stressors including staurosporine, MPTP and oxidative stress (Gegg et al., 2009, Haque et al., 2008, Wood-Kaczmar et al., 2008). Deregulation of mitochondrial calcium homeostasis is also associated with loss of PINK1 function (Abramov et al., 2011, Akundi et al., 2011, Gandhi et al., 2009, Heeman et al., 2011), suggesting that increased mitochondrial localization of PINK1 could lead to maintain functional interactions, activating channels and transporters along the mitochondrial network such as mPTP and NCXmito, to regulate calcium homeostasis and for instance, minimize potential excitotoxic effects. Very recently, a report has shown that PINK1 could prevent mitochondrial calcium overload through the activation of isoforms NCX2 and NCX3 (Wood-Kaczmar et al., 2013). Related to this, the Parkin mitochondrial localization enhances ER-mitochondria crosstalk, favoring the calcium transfer (Cali et al., 2013). Moreover, our results suggest that calcium overload could be necessary for the mitochondrial removal by PINK1/Parkin-mediated mitophagy (Narendra et al., 2008, Narendra et al., 2010b).

The identity of the transcription factor responsible for the increased *PINK1* transcription is unknown. FOXO3a increased PINK1 transcription in T-lymphocytes, MCF7 and SH-SY5Y cells upon growth factor/serum deprivation (Mei et al., 2009). *PINK1* transcription was also no longer increased following ischemia in FOXO1 and FOXO3 knock-out mice (Sengupta et al., 2011). However there was no evidence of FOXO3a increasing PINK1 transcription following treatment with CCCP. In this report, while we found that c-Fos translocated to the nucleus upon CCCP exposure, this had no effect on PINK1 protein levels. It is well-known that c-Fos is activated after pathological situations, such as hypoxia or ischemia (Cho et al., 2001), mediated by L- and N-type VDCCs (Premkumar et al., 2000, Zhao et al., 2007), so related to our results, c-Fos might be involved in adaptative responses upon calcium overload and mitochondrial depolarization promoting neuronal survival.

## Conclusions

**Fig. 12:**
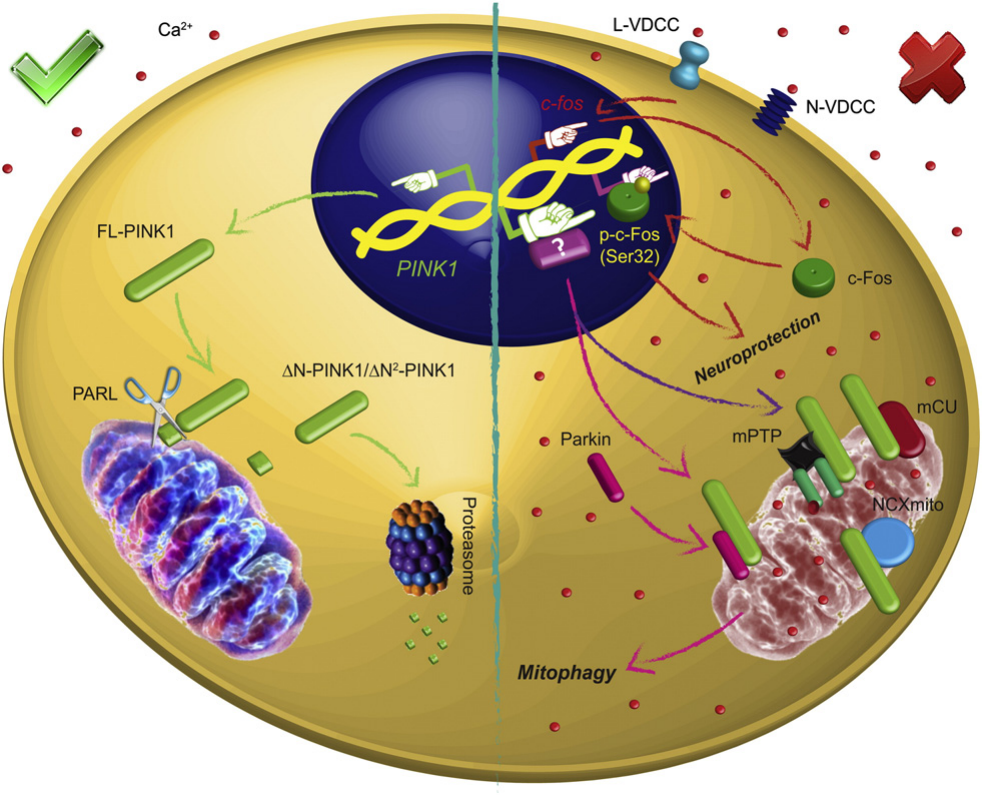
Schematic representation of CCCP effect in SH-SY5Y cells and the interplay between PINK1 and calcium. In physiological situations, Δψm is high, so FL-PINK1 is processed in mitochondria by proteases, including PARL. The cleaved isoforms are then degraded by UPS. However, when cells are exposed to CCCP, calcium influx through L- and N-type VDCCs increase *PINK1* gene expression, leading higher levels of PINK1 protein on damaged mitochondria, which might increase mitochondrial degradation by mitophagy (pink line) and/or modulate the activity of NCXmito, mPTP and/or mCU to control the mitochondrial calcium overload (purple line). This calcium influx also causes c-Fos activation, which probably translocates to the nucleus to promote neuroprotective signaling.

This study shows that treatment of SH-SY5Y cells with CCCP increases *PINK1* gene expression for at least 24 h, leading to a large accumulation of PINK1 protein on damaged mitochondria. This increase was calcium-dependent and might be via an influx of calcium via L- and N-type VDCCs, allowing for the mitochondrial removal by mitophagy. Moreover, the transcription factor c-Fos was activated by calcium, although was not responsible for induction of PINK1 transcription. The induction of PINK1 expression and activation of c-Fos could be a mechanism by which the cell activates neuroprotective mechanisms that might result in restoring calcium homeostasis and/or promote mitochondrial biogenesis (Fig. 12).

## Abbreviations

Act. D: actinomycin D
AP-1: activator protein-1
Baf. A1: bafilomycin A1
BAPTA-AM: 1,2-bis(2-aminophenoxy)ethane-N,N,N′,N′-tetraacetic acid acetoxymethyl ester
BSA: bovine serum albumin
c-Fos: FBJ murine osteosarcoma viral oncogene homolog
CCCP: carbonyl cyanide m-chlorophenylhydrazone
CHX: cycloheximide
CREB: cyclic AMP response element binding protein
CS: citrate synthase
Δψm: mitochondrial membrane potential
EGTA: ethylene-bis(oxyethylenenitrilo)tetraacetic acid tetrasodium
ER: endoplasmic reticulum
FCCP: carbonyl cyanide p-trifluoromethoxyphenylhydrazone
GAPDH: glyceraldehyde 3-phosphate dehydrogenase
Ho: Hoechst 33342
kDa: kilodaltons
LC3: microtubule-associated protein 1 light chain 3
mCU: mitochondrial uniporter
MG-132: carbobenzoxyl-leucyl-leucyl-leucinal
MPTP: 1-methyl-4-phenyl-1,2,3,6-tetrahydropyridine
mPTP: mitochondrial permeability transition pore
NCXmito: mitochondrial Na^+^/Ca^2 +^ exchanger
NF-κB: nuclear factor-κB
ω-CTX: ω-conotoxin GVIA
PARL: presenilin-associated rhomboid-like
PBS: phosphate buffered saline
PINK1: PTEN-induced kinase 1
PVDF: polyvinylidene fluoride
PI: propidium iodide
PD: Parkinson's disease
TMRM: tetramethylrhodamine methyl ester
TBST: Tris-buffered saline with Tween 20
UPS: ubiquitin-proteasome system
VDAC: voltage-dependent anion channel
VDCC: voltage-dependent calcium channel

The following are the supplementary data related to this article.

## Funding

This work was supported by FUNDESALUD [PRIS11014]; Gobierno de Extremadura [GR10054]; Instituto de Salud Carlos III [PI11/00040, PI12/02280, CB06/05/0041]; and Wellcome Trust/MRC Joint Call in Neurodegeneration award [WT089698] to the UK Parkinson's Disease Consortium (UKPDC). R.G-S. was supported by a FPU predoctoral fellowship (Ministerio de Educación, Spain), M.E.G. was supported by the Wellcome Trust/MRC Joint Call in Neurodegeneration award [WT089698] to UKPDC (United Kingdom), J.M.B-S. was supported by a postdoctoral contract (Ligue Nationale Contre le Cancer, France), M.N-S. was supported by a postdoctoral fellowship (Gobierno de Extremadura, Spain), L.A-E. was supported by a Senior Research Fellowship (Parkinson's UK, United Kingdom), E.P-E. was supported by a predoctoral fellowship (CIBERNED, Instituto de Salud Carlos III, Spain). RA.G-P. was supported by a “Miguel Servet” research contract (Instituto de Salud Carlos III, Spain).

## Conflict of interest statement

None declared.
